# Lignin‐Sourced Aromatics for Biodegradable Flexible Copolyesters Mimicking Poly(Butylene Adipate‐*co*‐ Terephthalate)

**DOI:** 10.1002/cssc.202501297

**Published:** 2025-10-16

**Authors:** Tam T. Nguyen, Maria Nelly Garcia Gonzalez, Jonas Engqvist, Jan Wahlberg, Gangjin Liu, Jing Liu, Patric Jannasch, Baozhong Zhang

**Affiliations:** ^1^ Centre for Analysis and Synthesis Department of Chemistry Lund University P.O. Box 124 SE‐221 00 Lund Sweden; ^2^ Environmental and Energy Systems Studies Department of Technology and Society Lund University SE‐221 00 Lund Sweden; ^3^ Division of Solid Mechanics Lund University P.O. Box 118 SE‐221 00 Lund Sweden; ^4^ Tetra Pak Packaging Solutions AB Ruben Rausings gata 2 223 55 Lund Sweden; ^5^ BPC Instruments AB Mobilvägen 10 SE‐223 62 Lund Sweden; ^6^ Division of Biotechnology Department of Chemistry Lund University P.O. Box 124 SE‐221 00 Lund Sweden

**Keywords:** biobased packaging, biodegradable plastics, bioplastics, lignin‐based polymers, sustainable polymers

## Abstract

Poly(butylene adipate‐*co*‐terephthalate) (PBAT) is an important commercial biodegradable flexible copolyester, which is dependent on the fossil‐based terephthalates for production. In the present work, two series of PBAT‐mimicking copolyesters are synthesized using lignin‐sourced aromatic monomers, i.e., methyl 4‐(2‐hydroxyethoxy) vanillate and methyl 4‐(2‐hydroxyethoxy) benzoate, aliphatic dimethyl adipate, and 1,4‐butanediol. The greenhouse gas emissions associated with the monomer synthesis are investigated by life cycle assessment, and the solvent usage is evaluated. The copolyesters show reasonably high thermal stability, and tunable glass transition temperature and crystallinity upon varying the aromatic–aliphatic ratio. Aerobic biodegradation experiments of the obtained copolyesters over 90 days show a comparable or even faster biodegradation rate compared to the benchmark polymer PBAT. The oxygen gas barrier of the obtained terephthalate‐free copolyester films is effectively enhanced compared to that of PBAT, indicating their potential in flexible food packaging applications.

## Graphical abstract

In this study, two series of novel PBAT‐mimicking copolyesters are introduced by exploiting lignin‐based building blocks. This class of copolyesters exhibits good barrier properties and biodegradability, paving the way for terephthalate‐free, biodegradable, flexible copolyesters.

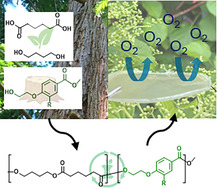

## 1 Introduction

Today, the wide use of polymers has been criticized because of the global environmental challenges they have caused, such as the depletion of fossil resources, plastic littering, microplastics, and greenhouse gas (GHG) emissions.^[^ ^1^, ^2^ ^]^ Therefore, developing biobased polymers from renewable resources with deliberately end‐of‐life properties (recyclability or biodegradability) has gained growing importance and attention.^[^ ^3^, ^4^, ^5^, ^6^ ^]^ Polyesters constitute a major class of bioplastics, which could be conveniently produced by using various bio‐sourced molecules with suitable functional groups (e.g., OH, COOH, and COOMe). Furthermore, bearing a polar main‐chain ester linkage allows the polymer to be susceptible to chemical or biological cleavage by multiple methods, thus promoting more end‐of‐life options for the material. Currently, most commercial polyesters (e.g., PET, PBT, etc.) contain aromatic building blocks, which endow desirable material properties for various applications (e.g., bottles, textiles, films, etc.), but they rely heavily on fossil‐based terephthalic acid (TPA) and are mostly nonbiodegradable (although there has been rapid development in the enzymes and microorganisms that degrade PET).^[^ ^7^, ^8^, ^9^, ^10^, ^11^, ^12^, ^13^ ^]^ Exploration on other biobased aromatic building blocks that can replace fossil‐based terephthalates for polyester production (mostly homopolyesters) has been an active and important research field, such as those based on furan,^[^ ^14^, ^15^, ^16^ ^]^ thiophene,^[^ ^17^ ^]^ indole,^[^ ^18^ ^]^ and various lignin‐based aromatics.^[^ ^19^, ^20^, ^21^ ^]^

Generally, incorporating aliphatic ester units into aromatic polyesters (namely aromatic–aliphatic copolyesters)^[^ ^22^, ^23^ ^]^ is an effective method to achieve balanced materials properties (e.g., thermal and mechanical) and biodegradability.^[^ ^6^ ^]^ The most important example is poly(butylene adipate*‐co*‐terephthalate) (PBAT), which is widely commercialized as biodegradable flexible films for agricultural and packaging applications.^[^ ^24^ ^]^ The structural design of PBAT offers flexibility and biodegradability through aliphatic segments (adipic acid and 1,4‐butanediol), while the aromatic segments (i.e., terephthalates) contribute to the thermal and mechanical quality. However, the aromatic segments of PBAT are still fossil‐based terephthalates, so the development of new PBAT‐type polyesters by using biobased aromatics is an important research topic. In contrast to the intensive studies on various biobased aromatics for homopolyesters, only a few biobased aromatics have been investigated so far in the development of PBAT‐type copolyesters, such as those based on 2,5‐furandicarboxylic acid (FDCA) and hydroxycinnamic units.^[^ ^25^, ^26^, ^27^, ^28^, ^29^, ^30^ ^]^ These new biobased PBAT‐analogous copolyesters can exhibit enhanced material properties (e.g., elasticity, oxygen barrier, and biodegradation rate) compared to PBAT,^[^ ^31^, ^32^, ^33^ ^]^ which show significant potential in various applications (e.g., food packaging) to improve sustainability.

Lignin is a particularly important biomass resource for aromatic building blocks and polymers.^[^ ^34^, ^35^, ^36^, ^37^ ^]^ Currently, only one lignin‐sourced aromatic molecule, vanillin, is commercialized, and two analogous aldehydes (4‐hydroxybenzaldehyde and syringaldehyde) are frequently found in the lignin depolymerization products in smaller amounts.^[^ ^38^ ^]^ In the meantime, various monoacids have also been investigated as lignin‐based molecules (e.g., homovanillic acid, syringic acid, and vanillic acid).^[^ ^39^, ^40^, ^41^, ^42^, ^43^, ^44^ ^]^ It has also been reported that 4‐hydroxybenzoic acid can be produced from lignin‐based *p*‐coumaric acid in near‐quantitative yield.^[^ ^45^ ^]^ The highly active research in lignin valorization has motivated the active exploration of biobased polymers using various lignin‐sourced aromatic molecules.^[^ ^5^ ^]^ During the past decade, many lignin‐sourced monomeric and dimeric molecules (e.g., vanillin,^[^ ^46^, ^47^, ^48^ ^]^ vanillic acid,^[^ ^49^, ^50^, ^51^ ^]^ syringaldehyde,^[^ ^52^, ^53^ ^]^ syringic acid,^[^ ^54^ ^]^ 4‐hydroxybenzoic acid,^[^ ^55^ ^]^ homovanillic acid,^[^ ^56^ ^]^ ferulic acid,^[^ ^57^, ^58^ ^]^ bis‐vanillin,^[^ ^59^ ^]^ and many other small molecular phenolics)^[^ ^60^ ^]^ have been extensively investigated to produce versatile sustainable polymers (e.g., thermoplastics, thermosets, and vitrimers) with a wide range of materials properties. Notably, while lignin and its substructures (e.g., vanillin, and vanillic acid) have been widely investigated as excellent gas barrier materials,^[^ ^61^, ^62^, ^63^, ^64^, ^65^ ^]^ it has been recently reported that certain lignin‐based aromatic monomer with spirocyclic acetal structures could effectively enhance the oxygen gas barrier properties for the polyester films.^[^ ^66^ ^]^

Investigations of lignin‐sourced aromatics for PBAT‐mimicking copolyesters remain limited. In 2013, Meier et al. reported the synthesis of low *T*_g_ aromatic–aliphatic copolyesters using potentially lignin‐sourced aromatic *p*‐hydroxycinnamic acids and rapeseed oil‐based aliphatic monomers.^[^ ^67^ ^]^ Later, copolymerizations of monomers based on aromatic vanillic acid and aliphatic unsaturated fatty acid (ricinoleic acid) or cyclic caprolactone have been reported, yielding biobased aromatic–aliphatic copolyesters with generally low molecular weight.^[^ ^54^, ^57^, ^68^ ^]^ Other low molecular weight aromatic–aliphatic copolyesters based on lignin‐sourced monomers have also been reported.^[^ ^69^, ^70^, ^71^ ^]^ Decent molecular weight was achieved later in 2021 by using an unsaturated aromatic monomer based on ferulic acid for the synthesis of aromatic–aliphatic copolyesters, which showed the ability to be crosslinked by electron beam irradiation.^[^ ^72^ ^]^ In 2023, lignin‐sourced coumarate/ferulate was used to replace terephthalates in PBAT, yielding the corresponding copolyesters with desirable chemical recyclability.^[^ ^30^ ^]^ In summary, the past decade has witnessed a growing interest in PBAT analogues using lignin‐based aromatics. However, the molecular design and the material performance, as well as their environmental impacts, largely remain to be investigated and developed.

**Scheme 1:**
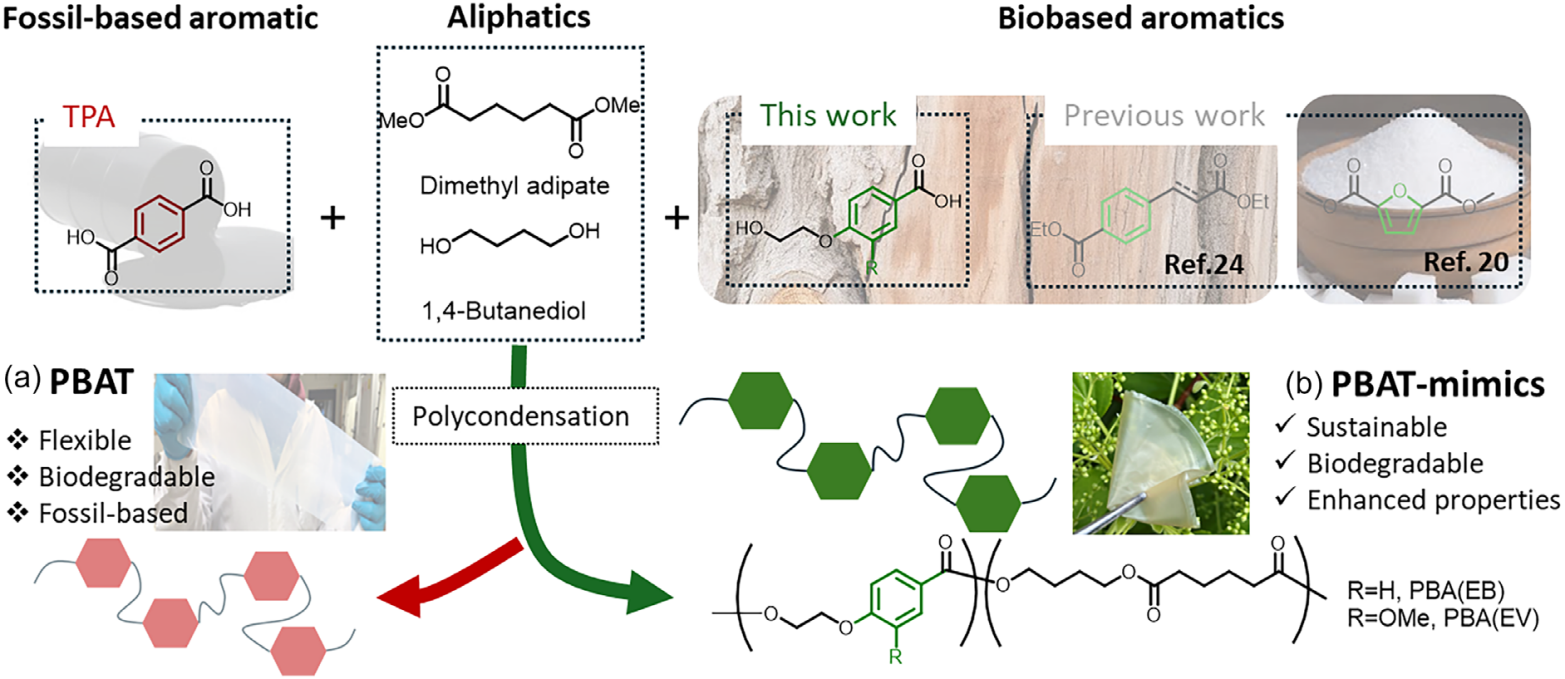
Synthesis and properties of a) commercialized PBAT and b) PBAT‐mimicking copolyesters from biobased aromatics in the previous work and our present work.

Herein, two novel series of terephthalate‐free copolyesters were synthesized using two lignin‐sourced aromatic monomers, methyl 4‐(2‐hydroxyethoxy) vanillate (MEV) and methyl 4‐(2‐hydroxyethoxy) benzoate (MEB) (**Scheme** **1**). The impact of the lignin‐sourced aromatics on thermal, mechanical, and oxygen barrier properties of the obtained copolyesters were investigated and compared with those of the commercial benchmark PBAT. The LCA perspectives regarding GHG emissions associated with the monomer synthesis as well as the aerobic biodegradation of the new PBAT‐mimicking copolyesters were also investigated for the first time.

## 2 Result and Discussion

### 2.1 Monomers Synthesis

**Figure 1:**
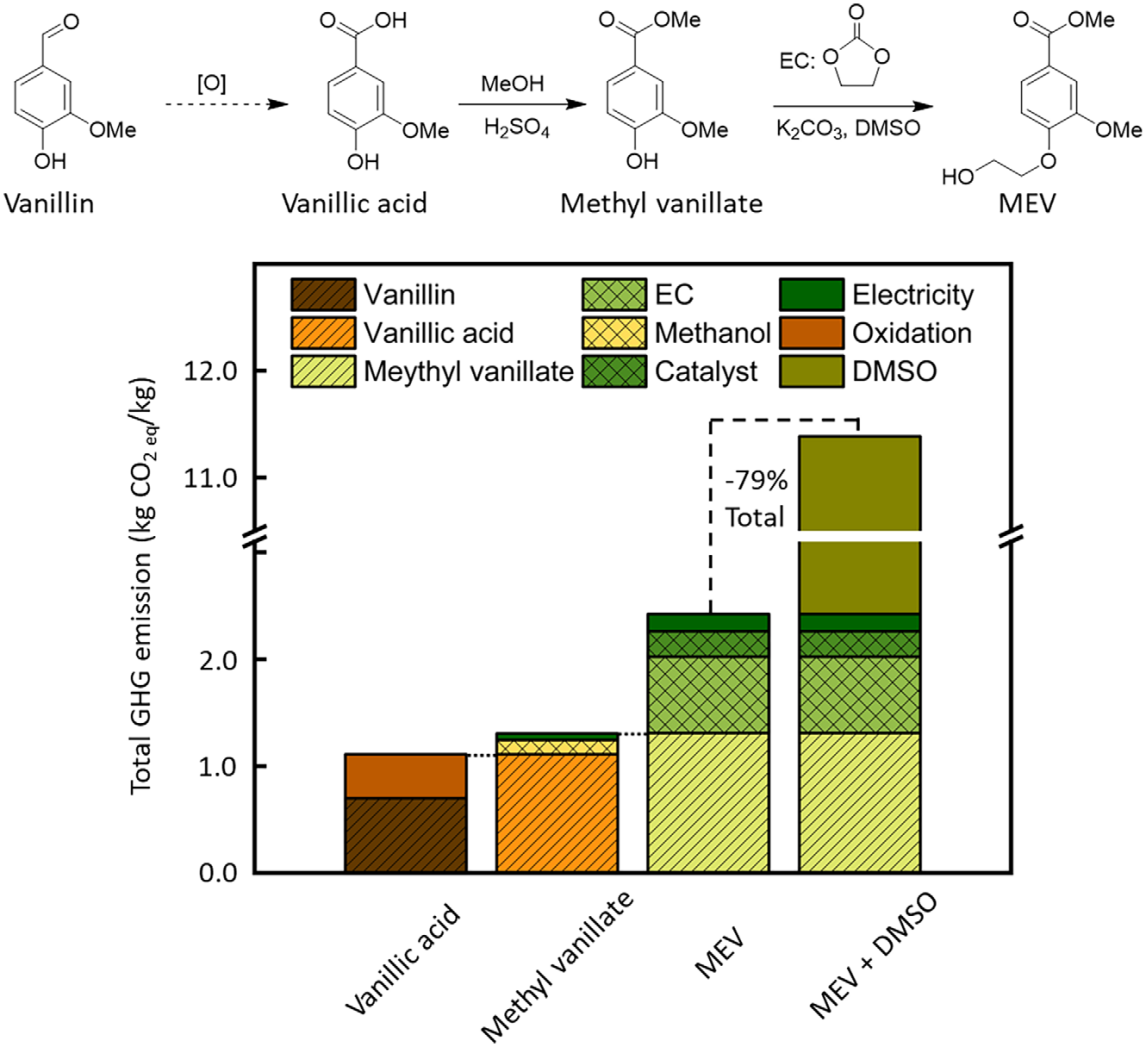
The schematic monomer synthesis, and the total GHG emissions of monomer MEV, both with and without the solvent DMSO, are divided into various contributions from the different steps.

Two AB‐type monomers, MEV and MEB, were synthesized using two lignin‐sourced molecules, vanillic acid and 4‐hydroxybenzoic acid, following a modified two‐step procedure (**Figure** **1**).^[^ ^56^ ^]^ First, the COOH groups in the starting molecules were converted into the corresponding methyl esters by a straightforward acid‐catalyzed esterification with methanol. Afterward, the obtained methyl vanillate (or methyl 4‐hydroxybenzoate) was reacted with 1 eq. of ethylene carbonate (EC) under mild basic conditions in DMSO at 100 °C for 5 h, giving the two monomers in satisfying yield (≈84% and 77% for MEB and MEV, respectively) after a straightforward purification by extraction. The purity and molecular structures of the two obtained monomers were confirmed by ^1^H, ^13^C, and 2D NMR spectroscopy (Figure S1–S6, Supporting Information).

### 2.2 Assessing the Environmental Impact of MEV Synthesis: E Factor, Atom Economy, and CO_2_‐Equivalent Emissions

A preliminary analysis of the life cycle GHG emissions was conducted as an early assessment of the bio based MEV monomer, focusing on the emissions associated with its lab‐scale synthesis (Figure 1). In general, the CO_2_‐equivalent GHG emissions for MEV synthesized from vanillin, oxidation to vanillic acid, then esterification to methyl vanillate and finally, alkylation to MEV were estimated as 11.4 kg of CO_2_‐eq kg^−1^ of MEV of which 13.5% was related to the raw materials including, vanillin, methanol, and EC (total 1.5 kg CO_2_‐eq kg^−1^ of MEV). Around 7.5% (total 0.9 kg CO_2_‐eq kg^−1^ of MEV) of total GHG emissions came from electricity and catalysts, including oxidation (3.5%), esterification (0.5%), and alkylation (3.5%). Finally, the production of the used solvent in the alkylation step (i.e., DMSO in our current lab‐scale procedure) accounted for 79% of the total GHG emissions. This revealed the significant impacts of the solvent on the GHG emissions, which should be a factor that needs to be carefully considered for optimizing the monomer synthesis procedure, particularly in an industrial scale, in the future.^[^ ^48^ ^]^

The synthesis of the two monomers was also evaluated regarding the environmental factor (E factor),^[^ ^73^ ^]^ and atom economy (AE).^[^ ^74^ ^]^ The calculation for the esterification and alkylation steps of MEV monomer synthesis followed the modified method reported in the literature,^[^ ^51^, ^56^ ^]^ and the details are presented in Table S2, Supporting Information. Regarding the alkylation reaction with EC, the monomer synthesis showed good AE values (76% and 81% for MEV and MEB, respectively) and relatively low simple E factor (sEF) (≈0.58 for both monomers) without considering the solvent (which is a commonly adopted procedure). When the solvent usage (i.e., DMSO) was considered, the complete E factor (cEF) values went up to 7.95 for MEV and 6.92 for MEB, of which ≈92% was due to the solvent contribution. The true E factor falls between the cEF and sEF, depending on solvent recovery and production scale in the following manufacturing stage.^[^ ^73^ ^]^

To select a more environmentally benign solvent for the MEV synthesis, different solvents have been screened (Table S1, Supporting Information). According to our results, the highest conversion of MEV was achieved when DMSO was used, and the amount of DMSO can be further reduced by a factor of four without any side effects. This demonstrated the potential for further optimization of monomer synthesis toward lower GHG emissions and reduced reaction waste.

### 2.3 Polymer Synthesis and Characterization

The two series of aromatic–aliphatic copolyesters were synthesized by using AB‐type aromatic monomers MEV or MEB and aliphatic monomers dimethyl adipate (DA) and 1,4‐butanediol (BDO) according to a two‐step polycondensation protocol (i.e., transesterification and polycondensation).^[^ ^20^, ^51^, ^56^, ^75^ ^]^ The first step (transesterification) was carried out at 130–150 °C for 4 h until all the monomers were completely consumed (monitored by ^1^H NMR). A continuous N_2_ flow was applied during this step to facilitate the removal of the by‐product methanol. During this step, partial sublimation of the monomer MEB was observed at 140–150 °C. This was consistent with the thermal gravimetric analysis (TGA) results, where weight loss above 144 °C was observed (Figure S7, Supporting Information). To avoid the monomer sublimation, the temperature for the transesterification of monomer MEB was kept at 130 °C. The second step (polycondensation) was carried out at a higher temperature (200 °C) under high vacuum (0.05 mbar) for 6 h. To obtain a reasonable molecular weight, an excess of BDO (diol) was usually required, presumably to compensate for the loss of BDO due to evaporation. The impact of the BDO excess was investigated by carrying out three polymerizations under the same conditions (time, temperature, and catalyst) with different amounts of BDO (i.e., 0, 5, and 12.5 mol% excess of BDO with respect to DA, Table S3, Supporting Information). The highest molecular weight was obtained when 5 mol% excess of BDO was used, which indicated the optimum amount for this reaction setup. According to this synthesis protocol, two series of in total eight copolyesters, including poly(butylene adipate) (PBA)(EV)_x_ series (using MEV, BDO, and DA) and PBA(EB)_y_ series (using MEB, BDO, and DA) with different chemical compositions (aliphatic/aromatic ratio), were successfully synthesized by varying the feed ratio between the monomers (**Table** **1**). The copolymers were named based on the feed ratio of aromatic monomers (i.e., MEV or MEB) to the aliphatic diester monomers (DA and BDO). For example, PBA(EV)_50_ is a copolyester with an equimolar amount of MEV and DA/BDO in the feed. According to size exclusion chromatography (SEC) measurement, reasonable molecular weights that allow for fundamental studies (*M*_n_ = 11.7–19.0 kg mol^−1^) were obtained (*Đ* ≈ 2.2–2.8). However, these numbers were lower compared to the molecular weight of the commercial PBAT sample (≈28.8 kg mol^−1^). Due to the insolubility in SEC solvent, the molecular weight of PBA(EV)_75_ copolymer was obtained via intrinsic viscosity (≈0.48 dL g^−1^), measured by an Ubbelohde viscometer using HFIP as solvent at 25 °C. As shown in Table 1, the content of aromatic units (i.e., EV or EB) in the final copolymers was slightly higher than in the feed ratio of comonomers. This could be attributed to the evaporation of PBA oligomers under high vacuum and at elevated temperatures during the polycondensation.

**Table 1:** Molecular weight and structure of the obtained copolymers.

PBA(EV)_X_	Feeda)	Composition [mol%]b)		Block lengthb)	*M*_n_ c) [kg mol^−1^]	*Đ* c)
	MEV(x):BDO/DA	EV	BA‐BDO	*L*_EV‐EV_		
PBA(EV)_25_	25:75	28	72	1.1	11.5	2.5
PBA(EV)_50_	50:50	56	44	1.7	18.4	2.4
PBA(EV)_60_	60:40	63	37	1.8	14.8	2.4
PBA(EV)_75_	75:25	77	23	2.8	18.5d)	–

PBA(EB)_Y_	MEB(y):BDO/DA	EB	BA‐BDO	*L*_EB‐EB_		
PBA(EB)_25_	25:75	27	73	1.2	15.7	2.2
PBA(EB)_50_	50:50	55	45	1.6	12.9	2.6
PBA(EB)_60_	60:40	66	34	2.0	17.1	2.8
PBA(EB)_75_	75:25	77	23	2.6	19.0	2.6

a) Polymerization condition: DBTO catalyst with a loading of 0.5 mol% to the total comonomer content, 130–150 °C under N_2_ flow for transesterification for 4 h, followed by a temperature ramp to 200–230 °C under vacuum for polycondensation over 6 h.

b) Calculated from ^1^H NMR: $L_{\text{EV}-\text{EV}}=I_{\text{EV}-\text{EV}}/I_{A-\text{EV}}+1,\;L_{\text{EBA}-\text{EBA}}=I_{\text{EBA}-\text{EBA}}/I_{A-\text{EBA}}+1$.

c) Determined by SEC in CHCl_3_.

d) PBAEV_75_The intrinsic viscosity [*η*] of 0.48 dL g^−1^ was measured with an Ubbelohde viscometer using HFIP as solvent at 25 °C, and *M*_v_ calculated by the Mark‐Houwink‐Sakurada equation using the parameters of PET in HFIP at 25 °C (*K* = 6.31 × 10^−4^ and *α* = 0.695).^[^ ^86^ ^]^

**Figure 2:**
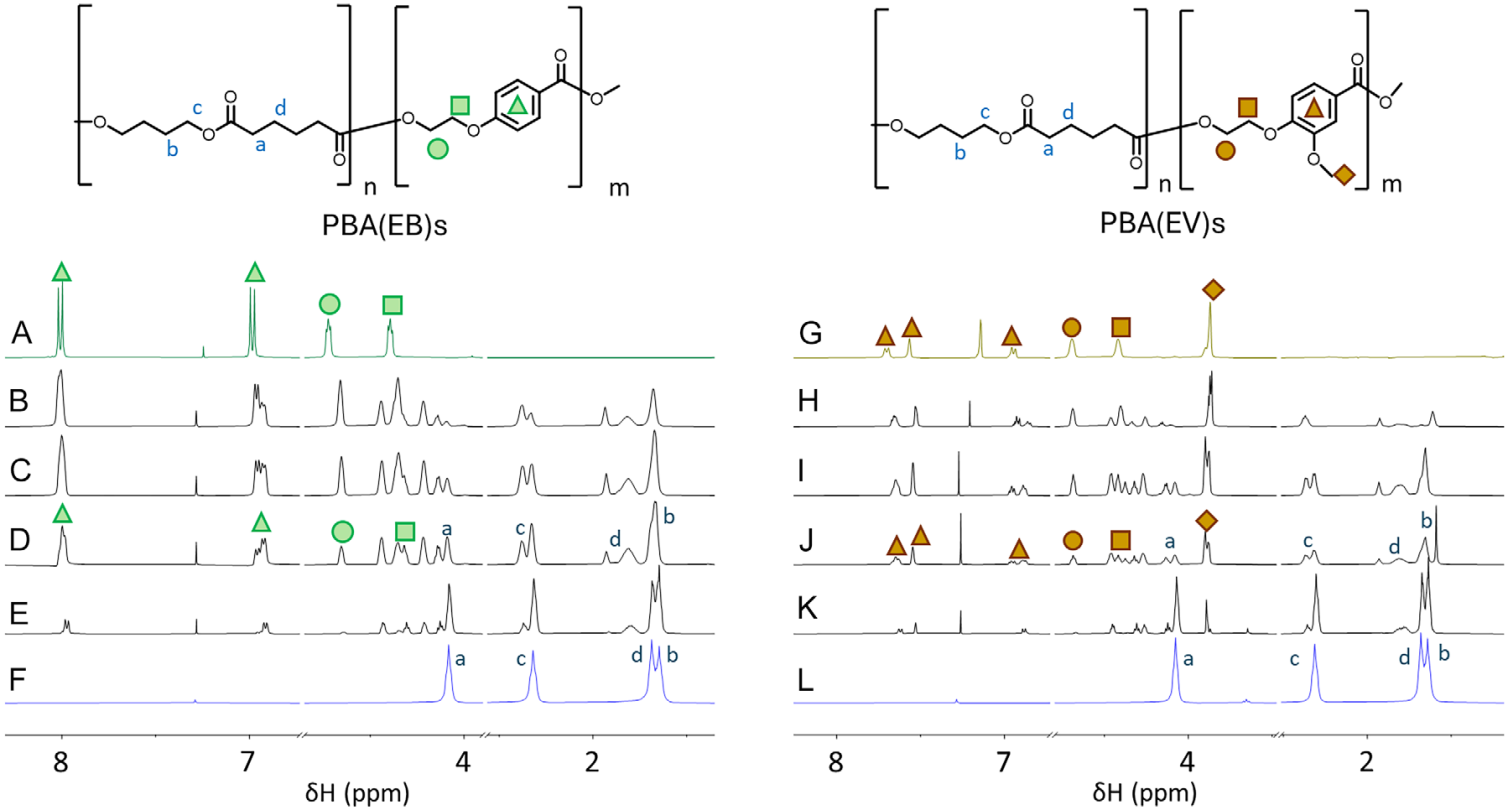
Selected regions of ^1^H NMR spectra of homopolymers A) Poly(ethylene oxybenzoate) (PEB), G) poly(ethylene vanillate) (PEV), and F,L) poly(butylene adipate) (PBA) and copolymers. B) PBA(EB)_75_, C) PBA(EB)_60_, D) PBA(EB)_50_, E) PBA(EB)_25_, H) PBA(EV)_75_, I) PBA(EV)_60_, J) PBA(EV)_50_, and K) PBA(EV)_25_ recorded in CDCl_3_, except PEB, PEV, and PBA(EV)_75_ which are record in CDCl_3_ and TFA‐d (8:2 v/v).

The molecular structures of the obtained copolymers were investigated by NMR spectroscopy. As shown in the ^1^H NMR spectra (**Figure** **2**), the signals corresponding to both the aliphatic units DA or BDO, and the aromatic units EV (or EB) were clearly observed in the copolymers, and their relative intensities were consistent with their chemical compositions. This confirmed the successful incorporation of the substructures in the copolymers. By inspection of the methylene region (4.0–5.0 ppm) in the ^1^H NMR spectrum (Figure 2D,J), typical signals originating from β‐methylene groups of BDO units (*d*), and α‐methylene groups of adipic units (*peaks a, a'*) were found in both copolyester series at ≈1.8 and 2.3 ppm, respectively. In the aromatic region, two signals from EB units were observed at 7.0 and 8.0 ppm for all the corresponding homo‐ and copolymers (Figure 2A–E). The incorporation of EV units in PBA(EV)s was confirmed by identifying the methoxy peak of the EV units at ≈3.9 ppm and the three corresponding aromatic signals at 6.9, 7.5, and 7.6 ppm (Figure 2G–K). The peaks corresponding to the ethylene spacer (i.e., OCH_2_CH_2_O units connected to benzene rings) were also observed at 4.2 to 5.0 ppm in both polymer series (Figure 2A–L). This clearly proved the formation of aromatic–aliphatic copolyesters, PBA(EV)s and PBA(EB)s with high purity.

Furthermore, the distribution of the aromatic monomeric units (e.g., EV or EB units along the backbone) was studied by 2D HMBC NMR spectra of PBA(EV)_50_ (Figure S12A–D, Supporting Information) and comparing NMR spectra of copolyesters with different compositions. As shown in Figure 2, the splitting for α‐methylene protons from EV and BDO units was observed in the ^1^H NMR region from 4.0 to 5.0 ppm due to the different ester‐dyads structures in the polymer backbone. The splitting was further confirmed by four distinct ^3^ *J* correlation spots between aromatic/aliphatic ester carbons and splitting α‐methylene proton signals of EV and BDO units in the 2D HMBC NMR spectrum of PBA(EV)‐50 (Figure S12B, Supporting Information). A similar effect was also observed in PBA(EB)_50_ NMR spectra (Figure S11, Supporting Information, green circle). With the intensities of the splitting methylene proton signals varying with comonomer composition, the average block lengths of EV and EB units were calculated and summarized in Table 1.^[^ ^66^ ^]^

### 2.4 Thermal Properties and Crystallinity

**Figure 3:**
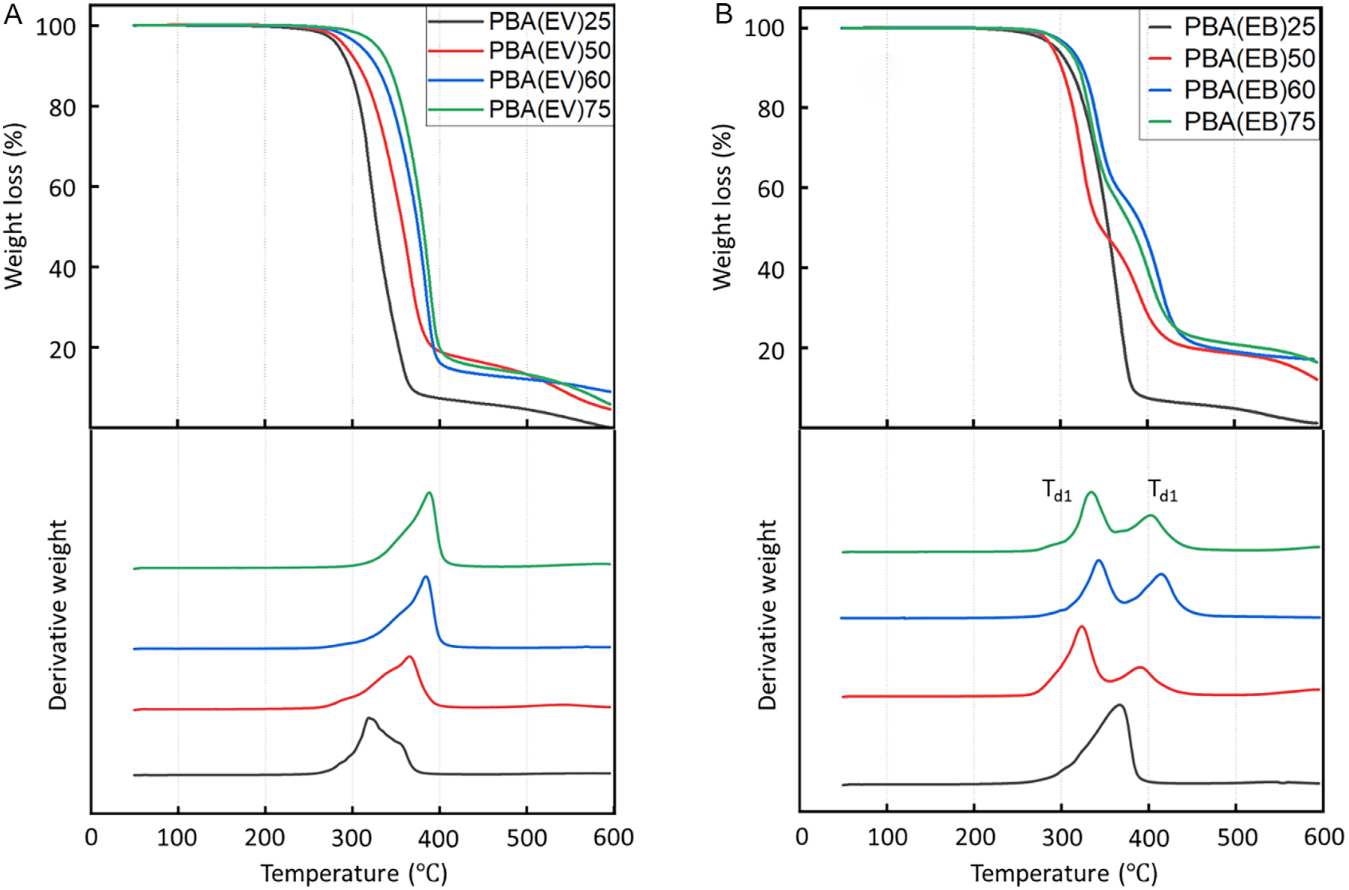
TGA curves of the weight loss and first‐derivative weight loss of A) PBA(EV)s and B) PBA(EB)s.

Thermogravimetric analysis (TGA), conducted under a nitrogen atmosphere, showed thermal decomposition onset temperatures (at 5% weight loss, *T*_d,5%_) typical for aliphatic–aromatic polyesters, ranging from 283 to 326 °C for PBA(EV)s, and 294 to 314 °C for PBA(EB)s. Within each series (i.e., PBA(EV) and PBA(EB)), the *T*_d,5%_ values were generally increased as the increased content of aromatic units (with the exception of PBA(EV)_60_, **Figure** **3**A), which was consistent with other reported copolyesters with aromatic and aliphatic compositions.^[^ ^30^, ^56^, ^76^ ^]^ Interestingly, two maximum degradation rate peaks of PBA(EB)s were observed in the derivative TGA curves (*T*_d_ in Figure 3B, **Table** **2**). The first thermal decomposition rate maxima were observed at around 321–343 °C, which was attributed to thermal degradation of the aliphatic ester linkage. The second maxima at around 392–414 °C was the typical thermal degradation temperature for aromatic ester linkage. In addition, the relative intensity of the two T_d_ values of each copolymer was consistent with the copolymers’ relative content of aromatic/aliphatic ester groups in the backbone.

**Table 2:** Thermal properties of the obtained copolymers.

PBA(EV)_X_	TGA		DSC						
			1st cycle		2nd heating			Annealingc)	
	*T*_d,5%_ a) [°C]	*T*_d,max_ b) [°C]	*T*_m1_^d)^ [°C]	*T*_c_ c) [°C]	*T*_g_ c) [°C]	*T*_cc_ c) [°C]	*T*_m2_ c) [°C]	Δ*H*_m_ [J g^−1^]	*X*_c,DSC_ [%]
PBA(EV)_25_	283	317	–	–	−33	–	–	–	–
PBA(EV)_50_	311	387	140	–	15	77	144	19.2	18.4
PBA(EV)_60_	306	351	161	–	28	74	164	30.2	29.0
PBA(EV)_75_	326	387	207	194	38	–	211	36.4	34.9

PBA(EB)_Y_									
PBA(EB)_25_	290	366	–	–	−37	–	–	–	–
PBA(EB)_50_	300	330, 402	–	–	−1	–	–	–	–
PBA(EB)_60_	305	340, 407	100	–	16	–	–	–	–
PBA(EB)_75_	314	343, 411	143	–	34	–	–	–	–
PBAT	360	400	–	90	−31	–	127	17.3	12.1

a) *T*_d,5%_ is the temperature with 5% mass in the TGA curves.

b) *T*_d,max_ is the temperature at which the maximum thermal decomposition rate during TGA measurement.

c) Samples were annealed at crystallization temperature.

The thermal properties of the obtained copolyesters were analyzed using differential scanning calorimetry (DSC), and the properties (e.g., melting points, degree of crystallinity, crystallization enthalpy, and glass transition temperatures) are shown in Table 2. According to the DSC second heating curves, the glass transition temperatures (*T*_g_) of all copolymers were in the range between the corresponding homopolymers, i.e., PBA (*T*_g_ ≈ −60 °C),^[^ ^77^ ^]^ PEV (*T*_g_ ≈ 75 °C), and poly(ethylene oxybenzoate) (PEB) (*T*_g_ ≈ 80 °C).^[^ ^20^, ^56^, ^78^ ^]^ Upon increasing the content of the aromatic units (i.e., EV or EB units), a clear increasing trend of *T*_g_ was observed in both PBA(EV) and PBA(EB) series, which was expected due to the enhanced rigidity of the backbone by the aromatic units. Furthermore, the PBA(EV) copolymers exhibited higher *T*_g_ compared to the corresponding PBA(EB) samples with the same aromatic content (Table 2). This was presumably due to the enhanced chain rigidity by the extra methoxy groups in the PBA(EV)s. This effect has been reported for other types of polymers.^[^ ^52^, ^79^, ^80^ ^]^ However, the two homopolymers (i.e., PEV and PEB) showed the opposite trend, as PEV with the additional methoxy groups showed slightly lower *T*_g_ (≈5 °C lower) than that of PEB. The exact reason(s) behind this observation remain to be explored, but it might be related to the extra free volume induced by the flexible methoxy groups.^[^ ^54^ ^]^ This has previously been reported for thermosetting and hyperbranched polymers.^[^ ^79^, ^81^, ^82^ ^]^

**Figure 4:**
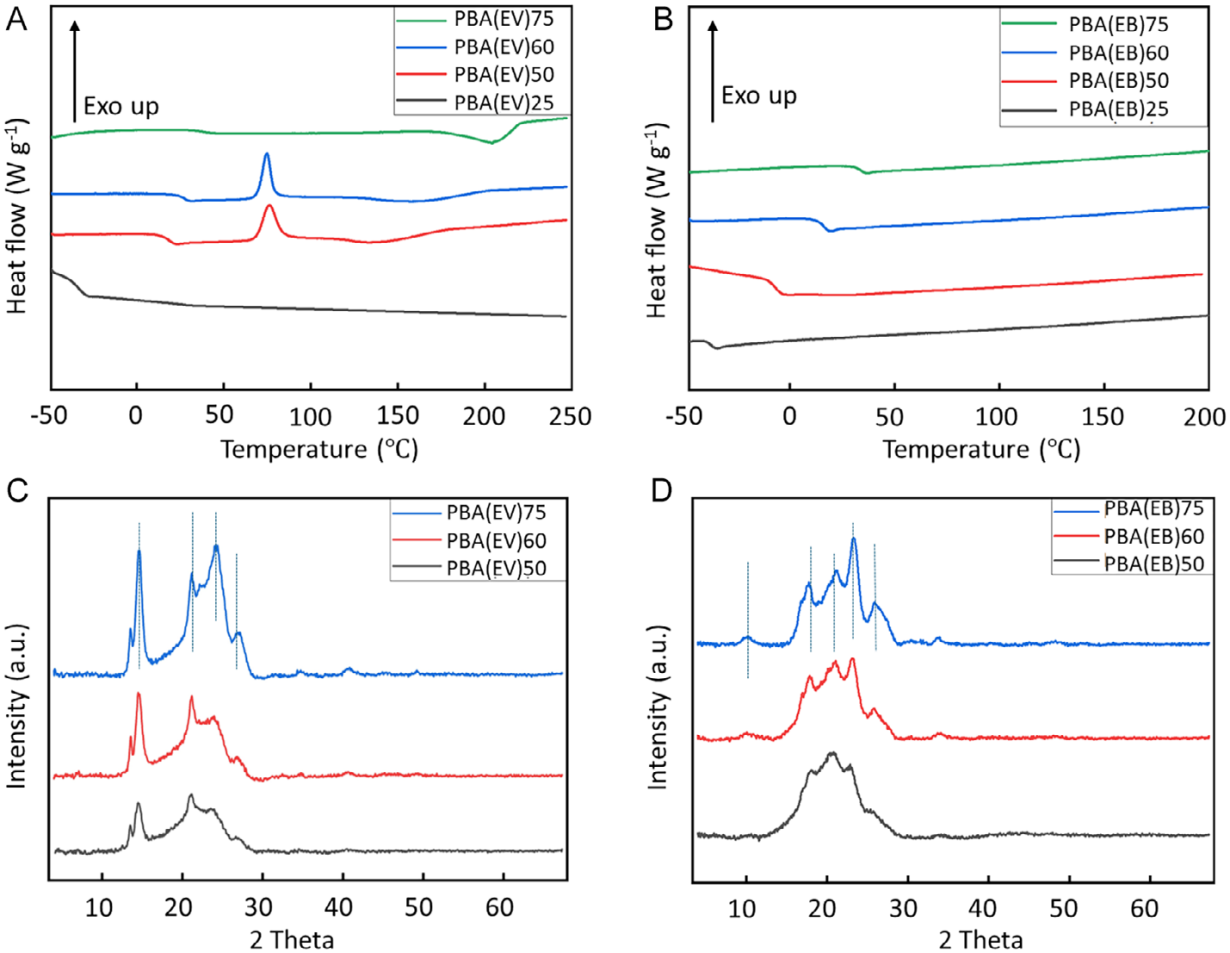
DSC second heating curves of A) PBA(EV)s and B) PBA(EB)s. WAXD patterns of the powder of C) PBA(EV)s and D) PBA(EB)s.

The incorporation of the two aromatic monomer units (i.e., EV or EB) showed significantly different impacts on the crystallization (**Figure** **4**A,B and Table 2). For the PBA(EV) series, the copolymers with ≥50% EV units showed crystallinity according to the DSC second heating curves. Broad melting peaks with an increasing *T*_m_ were observed at around 144 °C for PBA(EV)_50_, 164 °C for PBA(EV)_60_, and 211 °C for PBA(EV)_75_. Both PBA(EV)_50_ and PBA(EV)_60_ displayed cold‐crystallization behavior, which could be attributed to relatively low nucleation density as reported for polyethylene furanoate (PEF) and PEV.^[^ ^83^, ^84^, ^85^ ^]^ In contrast, the PBA(EB) copolymers were all completely amorphous, showing no melting peak during the second DSC heating cycle (Table 2). In this case, the presence of methoxy groups in the copolymer PBA(EV) series seems to enhance the interchain packing and thus crystallinity, although the molecular mechanism behind remains to be unraveled. In the literature, the crystallinity of polymers could be either enhanced or hindered by the presence of methoxy groups for different polymer systems.^[^ ^20^, ^21^, ^54^, ^57^ ^]^

To further understand the crystallinity, the obtained semicrystalline PBA(EV)s were thermally annealed at *T*_cc_ during the first cooling cycle, and then the maximum heat of fusion (Δ*H*_m_) and degree of crystallinity *X*_c,DSC_ were measured from the subsequent second heating cycle (Table 2). Both Δ*H*_m_ and *X*_c,DSC_ values increased with increased content of the aromatic EV segments.^[^ ^54^, ^84^ ^]^ For PBA(EB)s, when EB content < 75 mol% or *L*_EB‐EB_ < 2, the formation of crystalline structure is hindered, and this is consistent with other reported copolymers of MEB.^[^ ^56^ ^]^ As a result, all the obtained PBA(EB)s were amorphous according to the DSC second heating curves. However, the two copolyesters with relatively higher aromatic content, i.e., PBA(EB)_75_ and PBA(EB)_60_, showed melting endotherms during the first heating cycle, which showed that these two polymers could crystallize from their solutions (as precipitated powders) but not from the melt (under the DSC measurement conditions). Furthermore, no crystallinity could be observed even though the copolymer PBA(EB)_60–75_ was isothermally annealed at 80 °C.

To gain a better insight into the crystallinity of the obtained copolymers, the solution‐precipitated powders of PBA(EV)_50–75_ and PBA(EB)_50–75_ were subjected to wide angle X‐ray diffraction (WAXD) measurements (Figure 4C,D). PBA(EV)_25_ and PBA(EB)_25_ could not be measured because they had sticky and rubbery characteristics. For PBA(EV)_50–75_, similar diffraction peaks at 14.7°, 21.2°, 24.1°, and 27.0° were observed, which were consistent with the reported crystal structure of the corresponding aromatic homopolymer (i.e., PEV).^[^ ^84^ ^]^ The sharpness of the signals decreased from PBA(EV)_75_ to PBA(EV)_50_, which was consistent with the decreased crystallinity as observed in DSC results discussed earlier. For PBA(EB)_50–75_, WAXD patterns showed similar diffraction signals at 10.0°, 17.8°, 21.1°, and 26.0°, which were consistent with the peaks of the corresponding aromatic homopolymer (PEB).^[^ ^78^ ^]^ The sharpness of the observed signals also decreased from PBA(EB)_75_ to PBA(EB)_50_, which was consistent with the decreased crystallinity of the samples according to the 1^st^ heating cycle of DSC measurements. In the case of PBA(EB)_50_, no melting endotherm was observed in the DSC first heating cycle, but WAXD results indicated that the obtained powders contained a small extent of crystallinity, which was not measurable by DSC.^42,43^

### 2.5 Mechanical Properties

**Figure 5:**
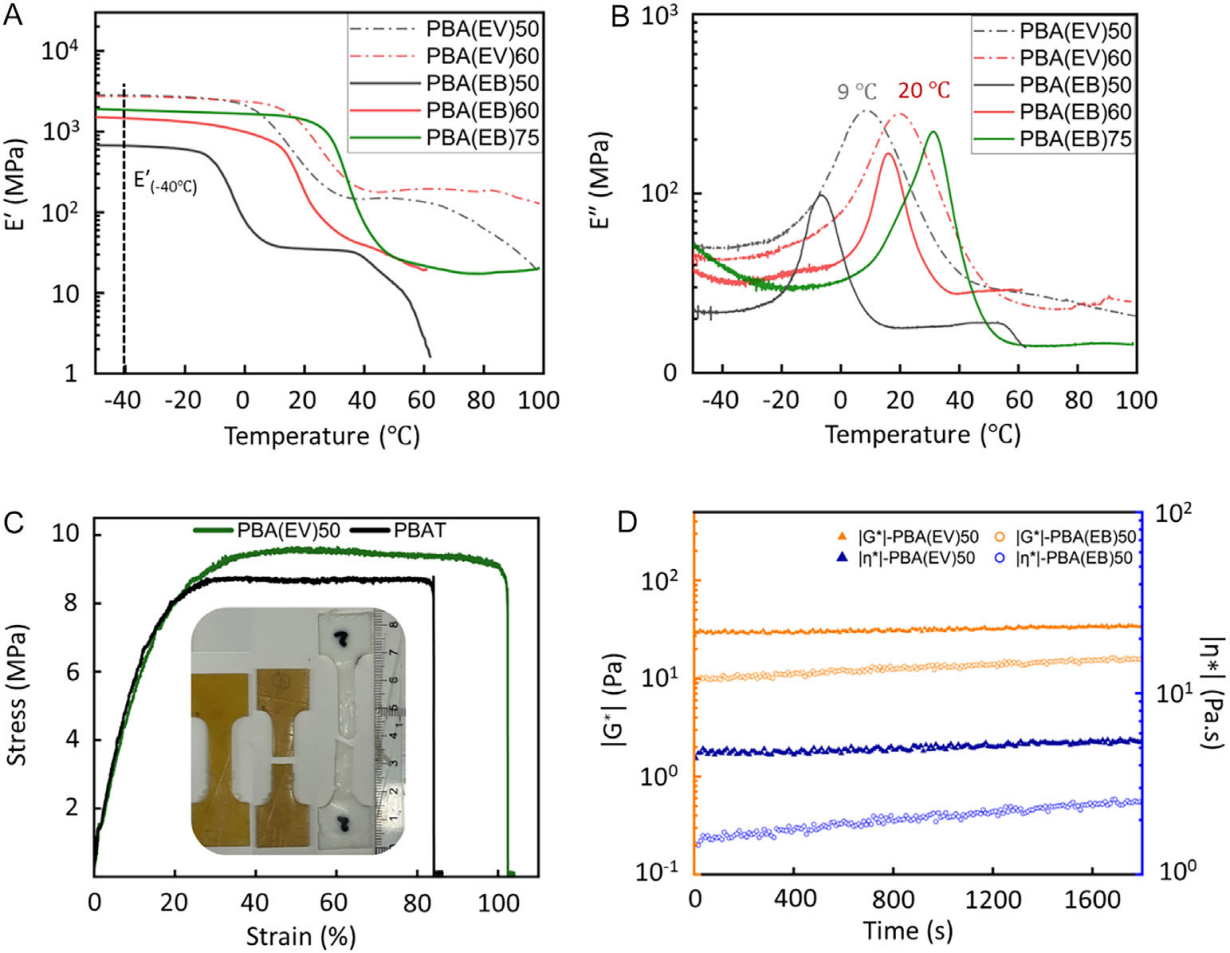
DMA data show A) storage and B) loss moduli for PBA(EV)s and PBA(EB)s copolyesters. C) Representative tensile stress–strain curves of PBAT and PBA(EV)_50_; and from left to right, typical dumbbell‐shaped specimen of PBA(EV)_50_, and tested dumbbell‐shaped specimens PBA(EV)_50_ and PBAT with *ε*_b_ ≈ around 100%. D) Dynamic melt rheology data of the complex dynamic shear modulus $\left|G^{*}\right|$ and complex viscosity $\left|\eta^{*}\right|$ for two copolyesters PBA(EV)_50_ and PBA(EB)_50_ at 200 °C over 30 min at 1% strain.

Dynamic mechanical analyzer (DMA) was performed for solid‐state experiments (**Figure** **5**A,B). Solid‐state samples for DMA were hot‐pressed into rectangular specimens (1.0 × 5.0 × 17.5 mm). While the specimens of amorphous PBA(EV)_25_ and PBA(EB)_25_ could not be produced due to the low *T*_g_, the PBA(EV)_75_ sample was too brittle for the mechanical measurement (due to insufficient molecular weight, probably below its entanglement molecular weight). The remaining copolyesters were successfully analyzed by DMA, and the temperature dependence from −50 to 100 °C of the storage (E') and loss moduli (E'') is reported in Figure 5A,B. The *T*_g_ values were taken as the local maxima of the loss moduli curves (E'', Figure 5B), which showed an increasing trend with increasing content of aromatic units for both the PBA(EV)s (*T*_g_ = 9 to 20 °C) and the PBA(EB)s (*T*_g_ = 16 to 32 °C). The *T*_g_ values obtained from the DMA data were slightly lower than those from DSC, i.e., 9 and 15 °C for PBA(EV)_50_, respectively. The reported E' values were taken at −40 °C, where all the copolymers were in a glassy state. Apparently, the PBA(EV)s possessed higher storage moduli (2.7–2.8 GPa) than those of PBA(EB)s (0.7 to 2.0 GPa) in the glassy plateau. This significant gap in storage moduli of the PBA(EV) specimens could be attributed to their crystallinity, which was absent in the hot‐pressed PBA(EB) specimens.

Tensile tests of PBA(EV)_50_ and PBA(EB)_50_ in comparison with PBAT were attempted using hot‐pressed molding dumbbell‐shaped specimens (2 × 8 mm in neck thickness and width). The tensile testing data of PBA(EB)_50_ specimens were unusable due to the softness of the low *T*_g_ (‐1 °C) copolymer (Figure S14, Supporting Information). As shown in Figure 5C and Table S4, Supporting Information, PBA(EV)_50_ showed a comparable Young's modulus (*E*) (56 MPa), ultimate tensile strength (*σ*_s_) (10 MPa), and elongation at break *ε*_b_ (97%) to PBAT (*E* ≈ 64 MPa, *σ*_s_ ≈ 9.6 MPa, and *ε*_b_ ≈ 89%) under the same measurement conditions. The relatively large error range of *ε*_b_ can be attributed to the imperfect sample preparation by the hot‐pressing method, which prevented nonuniform plastic deformation (necking). Finally, a difference in elastic recovery was observed in the broken specimens after around 100% stretching in the tensile testing (Figure 5C), where the PBA(EV)_50_ specimens showed instant elastic recovery, while the PBAT specimens suffered from irreversible deformation.

### 2.6 Dynamic Rheology

Dynamic rheology measurements were carried out to assess their stability and processability in the melt state. PBA(EV)_50_ and PBA(EB)_50_ were chosen as the representative samples for rheological measurement. Time sweeps of two copolyesters were carried out over 30 min with sinusoidal stress having a frequency of 1 Hz at 1% strain. Figure 5D shows the changes in complex dynamic shear modulus $\left|G^{*}\right|$ and complex viscosity $\left|\eta^{*}\right|$ of PBA(EV)_50_ and PBA(EB)_50_ at 200 °C over 30 min experiment. Both $\left|G^{*}\right|$ and $\left|\eta^{*}\right|$ of the two copolymers remained constant throughout the experiment. This result demonstrated the absence of significant degradation, such as crosslinking or chain scission, implying the melt processibility of the two copolyesters up to 200 °C.

### 2.7 Oxygen Barrier Properties

In this work, oxygen transmission rate (OTR) of the solution‐cast films of the obtained polyesters was measured to provide a firsthand insight into the structure–property relationship. Before OTR measurements, the powders of the polymer samples were cast into clear and homogeneous films (0.10 to 0.15 mm in thickness, 7.5 mm in diameter, Figure S13, Supporting Information) by a solution‐casting technique.^[^ ^47^, ^56^, ^66^ ^]^ The thermal properties and crystallinity of the obtained films were measured by DSC and WXRD (**Table** **3**) and compared to those of the corresponding polymer powders before film casting.

**Table 3:** OTR results of solvent‐cast films of the obtained copolyesters and commercial PBAT. The corresponding data of the powders (before film casting) are shown within parenthesis for comparison.

Samples	OTRa) [mL m^−2^ day^−1^, 0.21 atm]	*T*_g_ b) [°C]	*T*_m_ b) [°C]	Δ*H*_m_ b) (J g^−1^)	*X*_c,DSC_ c) [%]	*X*_c,XRD_ d) [%]
PBAT_50_	150.0	−32 (−31)	129 (127)	8.9 (17.3)	6.1 (12.1)	18.4
PBA(EV)_50_	25.8	15 (15)	147 (140)	11.2 (19.2)	10.8 (18.4)	24.5
PBA(EB)_50_	84.4	−1 (−1)	–	–	–	9.4
PBA(EB)_60_	29.6	16 (16)	115 (100)	5.5 (–)	6.3 (–)	28.7
PBA(EB)_75_	16.0	34 (34)	143 (143)	11.9 (–)	13.5 (–)	30.0

a) OTR; 23 °C, 50% relative humidity, 0.21 atm, the thickness was normalized to 100 μm for all the films.

b) Determined by DSC first heating scan from 0 to 250 °C of copolymer films with a heating rate of 10 °C min^−1^.

c) Calculated based on Δ*H*_m,PEV_^0^ = 104 J g^−1^; Δ*H*_m,PEB_^0^ = 88 J g^−1^; Δ*H*_m,PBT_^0^ = 142 J g^−1^.^[^ ^78^, ^84^, ^87^ ^]^

d) Calculated from the integrals of sharp WAXD signals of prepared films.

**Figure 6:**
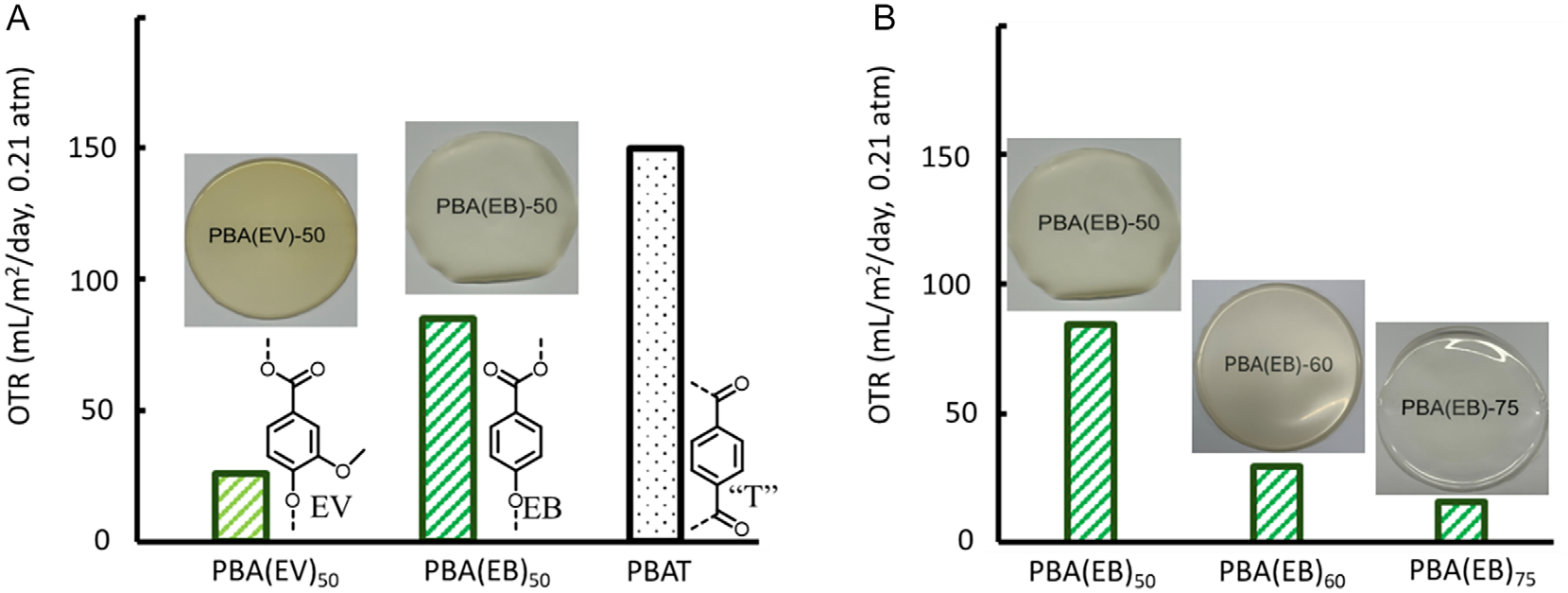
Oxygen transmission rate (OTR) of the obtained polymers and commercial PBAT, including A) three aromatic–aliphatic copolyesters with 50 mol% of the aromatics including the two biobased ones PBA(EV)_50_ and PBA(EB)_50_, and the benchmark PBAT (“*T*” denotes terephthalate units) and B) the same series of aromatic–aliphatic copolyesters with increasing content of the aromatics, EB.

As shown in **Figure** **6**, the measured OTR values of PBA(EV)_50_ and PBA(EB)_50_ films were significantly lower than those of the PBAT film of the same thickness, which preliminarily indicated that the incorporation of EV or EB units could enhance the oxygen barrier compared to terephthalate units (in all cases, 50% incorporation of aromatic units). This could be related to the greater rigidity of EV and EB monomeric building blocks compared to terephthalate units, as manifested by the higher *T*_g_ of PBA(EV)_50_ (≈15 °C) and PBA(EB)_50_ (≈−1 °C) to that of PBAT (≈−32 °C). Furthermore, the OTR value of PBA(EV)_50_ was significantly lower than that of PBA(EB)_50_ (≈70% lower), which suggested that EV units with methoxy substituents could enhance the oxygen barrier more than the EB units. Moreover, the higher crystallinity of PBA(EV)_50_ compared to those of PBA(EB)_50_ (Table 3) might also contribute to the observed lower OTR value of the former.

Furthermore, the effect of the aromatic content on OTR was evaluated by comparing the OTR values of three copolymers of the same series, i.e., PBA(EB)_50_, PBA(EB)_60_, and PBA(EB)_75_, with different aromatic/aliphatic ratios. As shown in Table 3 and Figure 6, it was clear that an increased content of aromatic EB units in the copolyesters enhanced the oxygen barrier. By incorporating 25% more EB units, i.e., from PBA(EB)_50_ to PBA(EB)_75_, the OTR value was lowered by ≈80%. This further confirmed the effectiveness of the aromatic EB structure in enhancing the oxygen barrier of the polymer films. Unfortunately, the other copolymers encountered problems in OTR measurements. PBA(EV)_60_ and PBA(EV)_75_ failed in OTR measurements, likely due to their higher crystallinity which could cause microsized cracks and holes during OTR measurements. PBA(EV)_25_ and PBA(EB)_25_ also failed because their corresponding films were too soft and sticky (as reflected by the low *T*_g_) to be measured. In summary, the observed better gas barrier properties of the obtained polymer films could have the potential to solve the inherent shortcoming in barrier properties of PBAT films for packaging applications, particularly food‐related products.

### 2.8 Aerobic Biodegradation under Composting Environment

**Figure 7:**
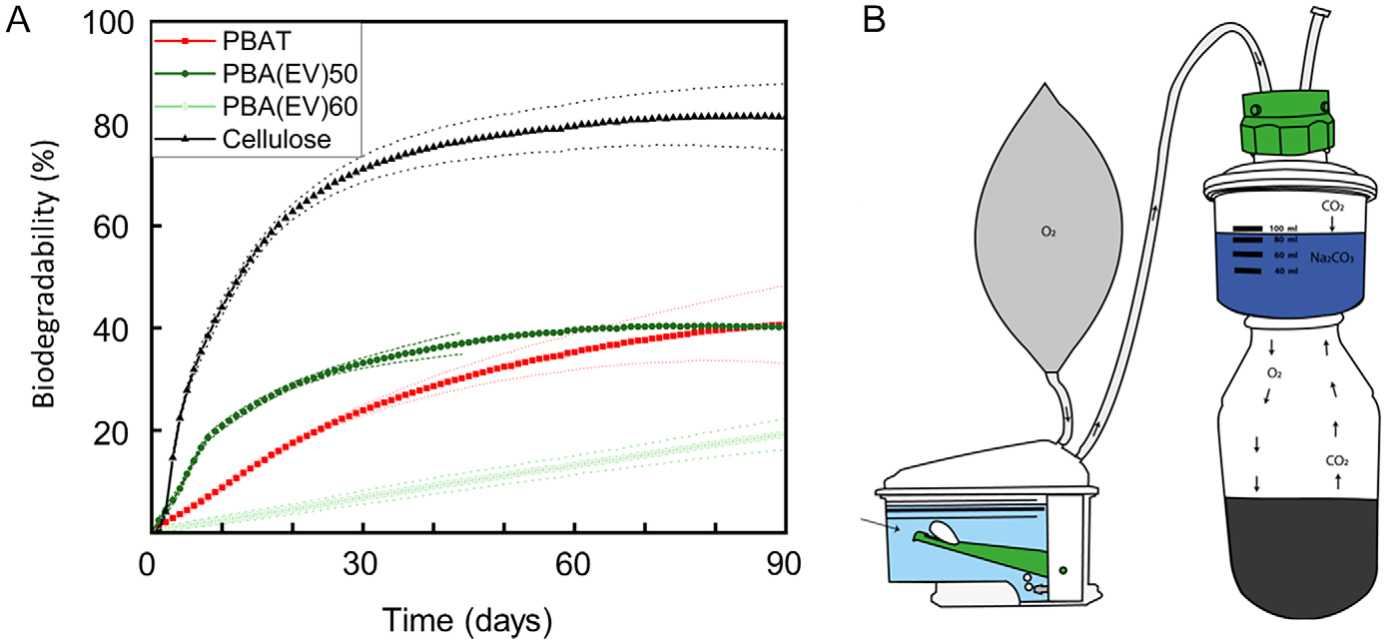
A) Biodegradation under composting environment of the two copolyesters, PBA(EV)_50_ and PBA(EV)_60_, as compared to microcrystalline cellulose and PBAT as reference materials and B) a scheme of the experimental setup for the aerobic biodegradation test at BPC Instruments AB.

Aerobic biodegradation test of as‐received polymer powders under composting environment (58 ±2 °C) was performed using a setup shown in **Figure** **7**B, in which the consumption of O_2_ by the test mixture was monitored in real‐time. The test result was validated based on ISO 14855‐1 according to the reference material biodegradation rate and the deviation among the triplicates. As shown in Figure 7A, PBA(EV)_50_ degraded faster than PBAT_50_ at the beginning, reaching 25.3% biodegradation after 15 days, while PBAT_50_ only reached 13.5% biodegradation. However, as the measurement continued, both samples reached a plateau at ≈40% biodegradation, which took ≈60 days for PBA(EV)_50_ and 84 days for PBAT_50_ to reach the plateau. Interestingly, the biodegradability and biodegradation rate were significantly lower for PBA(EV)_60_, with only 10% increased in aromatic content compared to PBA(EV)_50_. According to our results, PBA(EV)_60_ showed only 3.5% degradation after 15 days and ≈20% after 90 days. This slow biodegradation of PBA(EV)_60_ could be related to its higher crystallinity (≈29.0% vs. 18.4%, Table 2) as compared to PBA(EV)_50_. In the literature, a slower biodegradation rate due to increased crystallinity has also been reported for other aromatic–aliphatic copolyesters.^[^ ^27^ ^]^ Our results revealed that the biodegradation rate of the aromatic–aliphatic copolyesters was greatly influenced by the exact molecular structures, degree of crystallinity, hydrophilic–hydrophobic balance, and it was possible to achieve a comparable or even a faster biodegradation rate than PBAT by using the terephthalate‐free lignin‐sourced aromatics.

## 3 Conclusions

In this work, we reported two novel series of terephthalate‐free copolyesters using lignin‐based aromatic monomers via polycondensations with an aliphatic diol and an aliphatic dicarboxylate ester. The obtained copolyesters are fully biobased, which can be considered analogs to the commercial benchmark fossil‐based PBAT. The copolyesters displayed a fair thermal stability (*T*_d,5%_ ≈ 300 °C) with tunable thermal properties (*T*_g_ and *T*_m_) depending on the molecular structure and composition. The OTR results showed that the lignin‐sourced aromatic units were effective in enhancing the oxygen barrier for the polymer films compared to that of commercial PBAT, which indicated their potential in food packaging. Finally, copolyester PBA(EV)_50_ exhibited a comparable or even faster biodegradation rate under composting conditions. Future work will include further optimization of the polymerization parameters, preferably on a larger scale, which is expected to provide samples and valuable information for the further evaluation of the materials production, application, and sustainability‐related aspects.

## Conflict of Interest

The authors declare no conflict of interest.

## Author Contributions

**Tam T. Nguyen** conceived the project, performed the polymer synthesis and evaluations, and wrote the first draft of the manuscript. **Maria Nelly Garcia Gonzale**z performed LCA calculations. **Jonas Engqvist** conducted tensile testing. **Jan Wahlberg** contributed to the measurement of gas barrier properties. **Gangjin Liu** and **Jing Liu** carried out biodegradability measurements. **Patric Jannasch** and **Baozhong Zhang** conceived and supervised the project, provided funding, and revised the draft.

## Supporting information

Supplementary Material

## Data Availability

The data that support the findings of this study are available in the supplementary material of this article.
