# Supplementary material for: Lignin‐Sourced Aromatics for Biodegradable Flexible Copolyesters Mimicking Poly(Butylene Adipate‐co‐ Terephthalate)
Source: ChemSusChem. 2025 Oct 16;18(23):e202501297. doi: 10.1002/cssc.202501297 (PMC12665884; doi:10.1002/cssc.202501297)
Supplement: Supplementary file 1 — Supplementary Material [file CSSC-18-e202501297-s001.pdf]

Supporting Information

Lignin-Sourced Aromatics for Biodegradable Flexible Copolyesters Mimicking Poly(Butylene Adipate Terephthalate)

Tam T. Nguyen,[a] Maria Nelly Garcia Gonzalez,[b] Jonas Engqvist,[c] Jan Wahlberg,[d] Gangjin Liu,[e] Jing Liu,[e,f] Patric Jannasch,[,a] Baozhong Zhang*[,a]*

[a] Centre for Analysis and Synthesis, Department of Chemistry, Lund University, P.O. Box 124, SE-221 00 Lund, Sweden

[b] Environmental and Energy Systems Studies, Department of Technology and Society, Lund University, SE-221 00 Lund, Sweden.

[c] Division of Solid Mechanics, Lund University, P.O. Box 118, SE-221 00 Lund, Sweden.

[d] Tetra Pak Packaging Solutions AB, Ruben Rausings gata 2, 223 55 Lund, Sweden.

[e] BPC Instruments AB, Mobilvägen 10, SE-223 62 Lund, Sweden.

[f] Division of Biotechnology, Department of Chemistry, Lund University, P.O. Box 124, SE-221 00 Lund, Sweden

Corresponding Authors:

Baozhong Zhang. Email: baozhong.zhang@chem.lu.se

Patric Jannasch. Email: patric.jannasch@chem.lu.se

List of content

Materials

Analytical Methods

NMR analysis of monomers and polymer samples

Size exclusion chromatography

Life cycle assessment (LCA) methodology of GHG emissions

Thermogravimetric analysis (TGA)

Differential scanning calorimetry (DSC)

Wide angle X-ray diffraction (WAXD)

Dynamic mechanical analysis (DMA)

Rheology

Oxygen transmission rate (OTR)

Aerobic biodegradation evaluation

Monomer synthesis

Synthesis of methyl vanillate and methyl 4-hydroxybenzoate

Synthesis of methyl 4-(2-hydroxyethoxy) benzoate [MEB] monomer

Synthesis of the methyl 4-(2-hydroxyethoxy) vanillate [MEV] monomer

Polymer synthesis

Poly(1,4-butylene adipate-co-4-(2-hydroxyethoxy) vanillate) [PBA(EV)] and Poly(1,4-butylene adipate-co-4-(2-hydroxyethoxy) benzoate) [PBA(EB)]

Characterization of the monomers, MEV and MEB: Figure S1. - S7.

Characterization of the copolyesters, PBA(EV)s and PBA(EB)s: Figure S8. - S12.

Solution-casting film: Figure S13.

Tensile testing: Figure S14.

Table S1. Solvent screening for the synthesis of monomer MEV.

Table S2. Green metric calculations of monomer syntheses.

Table S3. Effect of initial excess of diol on the polymerization of $PBA(EV)_{50}$.

Table S4. Summary of tensile properties for the obtained copolyesters compared to that of PBAT.

Materials

Vanillic acid (97%), 4-hydroxybenzoic acid (99%), ethylene carbonate (98%), dimethyl adipate (DA, >99%), 1,4-butanediol (BDO, 99%), sulfuric acid (H_2SO_4), dibutyltin oxide (DBTO) (>98%), potassium carbonate (K_2CO_3, >99%), 1,8-diazabicyclo[5.4.0]undec-7-ene (DBU, 98%), 18-crown-6 (>99%), dimethyl sulfoxide (DMSO, ≥99.5%), dichloromethane (DCM, 99.8%), sodium bicarbonate ($NaHCO_3$), and sodium hydroxide (NaOH, pellets) were purchased from Sigma-Aldrich. Methanol (>99.8%) was purchased from VWR Chemicals and Sodium sulfate (Na_2SO_4, 99%) was supplied by Honeywell. 1,1,1,3,3,3-Hexafluoro-2-propanol (HFIP) was purchased from Nanjing Confidence Chemical Co., Ltd. Ecoflex® poly(butylene terephthalate) (PBAT) was obtained from BASF. All chemicals and reagents were used as received without further purification.

Analytical Methods

NMR analysis of monomers and polymer samples

^{1}H and ^{13}C nuclear magnetic resonance (NMR) measurements were performed on a Bruker DRX400 spectrometer at the proton frequency of 400.13 MHz and a carbon frequency of 100.61 MHz, respectively. Chemical shifts were reported as δ values (ppm).

Size exclusion chromatography

Size exclusion chromatography (SEC) measurements were carried out with Malvern OMNISEC instrument equipped with a TGuard, Org Guard, col 10 × 4.6 mm as a guard column, 2xT6000M, general mixed Org.300×8.0 mm as an analytical column, and a refractive index (RI) detector. All measurements were carried out at 35 °C with chloroform as the eluent and an elution rate of 1 mL min^{-1}. Calibration was performed with polystyrene standards (M_n= 96, 52.4, 30, 17.5, 3.5, and 3.0 kg mol^{-1}).

Life cycle assessment (LCA) methodology of GHG emissions

The characterization factors needed to quantify how much impact the new monomer (MEV) has on GHG emissions were taken from the IPCC 2021 method. We calculated the GHG emissions of MEV monomer based on our lab-scale synthesis route, which started with vanillic acid. Note that only monomer MEV was investigated in this preliminary LCA study because its potential precursor, vanillin (can be converted into vanillic acid), is the only commercial lignin-sourced aromatic chemical so far. For the other monomer MEB (4-hydroxybenzoic acid-based structure), there was no industrial scale production process nor LCA data available for the industrial production of the possible precursors (e.g., 4-hydroxybenzoic acid or methylparaben) from lignin sources, although LCA data based on hypothetical industrial-scale biosynthesis of 4-hydroxybenzoic acid using sugar resources have been reported.[43]

The energy required to produce 1 kg of methyl vanillate via the esterification step of vanillic acid was assumed to be equal to the energy required to produce 1 kg of fatty acid methyl esters (ref Ecoinvent database). The excess methanol used in the esterification step was assumed to be recovered. In the alkylation step, the energy required to produce 1 kg of monomer MEV via alkylation was assumed to be equal to the energy required to produce 1 kg of polyurethane at 100 °C (ref Ecoinvent database). The energy costs and production of reagents were also taken from the Ecoinvent database, version 3.9. The calculations were performed with and without considering DMSO as the solvent in the alkylation step. Transportation of feedstock and chemicals from one factory to the others for MEV production was excluded.

The GHG emissions of vanillic acid were estimated from the only commercially available lignin-derived molecule, vanillin. The climate change result of vanillin was taken from

Borregaard (0.7 kg CO_2 - eq/kg dry matter content).[44] To this value, the estimated GHG emissions corresponding to the process of oxidizing vanillin to vanillic acid were added. Due to the unavailability of life-cycle inventory data for the exact oxidation of vanillin to vanillic acid in the database, the GHG emissions for the oxidation of a similar molecule, toluene, into benzoic acid was used as an approximation (0.41 kg CO_2 - eq/kg molecule). The total GHG emissions associated with the production of vanillic acid were therefore estimated as 1.11 kg CO2 -eq/kg vanillic acid.

Thermogravimetric analysis (TGA)

The onset thermal decomposition temperature ($T_{d,5\%}$) and the maximum decomposition rate temperature ($T_{d,max}$) of dried powder copolymer were measured using a thermogravimetric analyser TA Instruments Q500. The samples were heated under a nitrogen atmosphere (50 mL min^{-1}) from 50 to 600 °C at a rate of 10 °C min^{-1}. $T_{d,5\%}$ was the temperature at which a 5% mass loss of the sample was observed, while $T_{d,max}$ was found at the maximum in the first derivative curves.

Differential scanning calorimetry (DSC)

DSC measurements were performed using a TA Instruments DSC Q2000. The samples were analyzed with a heating rate of 10 °C min^{-1} under nitrogen at a purge rate of 50 mL min^{-1}. The sequence consisted of a heating ramp from 0 °C to 250 °C, followed by a cooling ramp to -50 °C and finally a heating ramp to 250 °C. The glass transition temperature (T_g), the melting temperature (T_m), and the heat of fusion (ΔH_m) were determined from the second heating cycle. Isothermal crystallizations from the melt were studied at T_{cc} or T_c temperatures from 75-90 °C. The copolymers first melted at 250 °C for 5 mins and then cooled at a rate of 20 °C min^{-1} to the desired crystallization temperature. The temperature was then kept isothermally for 2 hours, followed by a heat ramp to 250 °C at a rate of 10 °C min^{-1}. The

melting point (T_m) and maximum heat of fusion (ΔH_m) were determined in the final heating cycle. The degree of crystallinity, $X_{c,DSC}$, was calculated from the ΔH_m^0 of homopolymers: PEV (104 J g^{-1}), PEB (88 J g^{-1}), and PBT (142 J g^{-1}).

Wide angle X-ray diffraction (WAXD)

WAXD measurements were performed using a Stoe STADI MP X-ray powder diffractometer under ambient conditions for copolymers in the powder and the film form. Measurements were performed over 2θ ranges of 5-60° with copper K_α (0.15406 nm) radiation. The degree of crystallinity, $X_{c,XRD,}$ was quantified based on the integrals of WAXD signals according to:

$$X_{c,XRD} = \frac{A_c}{A_c + A_m} * 100\%$$

Where A_c represents the area under crystalline diffraction patterns and A_m is the area under amorphous diffraction patterns.

Dynamic mechanical analysis (DMA)

DMA was performed using a TA instrument Q800 analyzer from -50 to 100 °C at a heating rate of 2 °C/min^{-1} and frequency of 1 Hz. A strain sweep was run for all samples, and the linear viscoelastic region (LVR) was determined. A strain of 0.05% was chosen within LVR and was used for all analyses.

DMA samples were prepared by first drying all polymer powders at 50 °C in a vacuum oven for 3 days before hot-pressing in a rectangular 1.0 × 5.0 × 17.5 mm mould. The hot-pressing processes were performed at 30 °C above their respective melting temperatures or 40 °C above the respective glass transition temperature.

Rheology

Rheological experiments were conducted on an AR2000 ETC Advanced Rheometer from TA Instruments. The measurements were performed in an oscillatory shear mode using a 25 mm diameter parallel plate geometry. A 30-minute time sweep was performed on all samples at 200 °C at a frequency of 1 Hz and 1% strain, which was within the linear viscoelastic region. Then, dynamic frequency sweeps between 0.01 and 100 Hz were recorded at 10% strain and 200 °C. Samples of 1 mm thickness were hot-pressed in a 25 mm circular mould.

Oxygen transmission rate (OTR)

OTR analysis was carried out by employing standard conditions at 23 °C with 21% O_2 and 50% RH with a partial pressure of 0.21 atm on an Ox-Tran 2/21 instrument from Mocon (now Ametek). The samples for OTR measurements were prepared according to a solution-casting protocol. 1 g of polymer powder was dissolved in 10 mL $CHCl_3$ at room temperature to yield a clear, viscous solution before evenly casting in Telfon crucibles (diameter of 75 mm). Then, the solvent was allowed to evaporate at room temperature under a glass conical funnel for 7 days to form a uniform film. The films were kept in the open air for 21 days before any measurements to ensure the complete removal of solvent.

Aerobic biodegradation evaluation

The ultimate aerobic biodegradability was performed to evaluate the biodegradability of the polymers under controlled composting conditions at 58 (±2) °C by a BPC Blue respirometer (BPC Instruments AB, Lund, Sweden), referring to the ISO 14855-1 and ASTM D5929 standards. Polymer samples were ground into a fine powder and dried thoroughly in a vacuum oven at 50 °C for at least 3 days before the test. The compost with a particle size of around 2 mm was obtained from NSR AB (Helsingborg, Sweden).

Briefly, the test samples were prepared by mixing 78.7 g of wet compost (50 g dry compost) with 10 g of vermiculite, before adding 2.0 g of polymer powder. The mixture was

thoroughly mixed, and the water content was regulated at 50%. The sample mixture was then loaded into a 1 L closed bottle connected to a scrubber vessel with 100 mL 3 M NaOH solution with thymolphthalein indicator to trap generated CO_2. Each polymer sample (including blanks and reference material cellulose) is prepared in triplicate. To start the measurement, the testing bottle was installed in an air incubator at 58 ℃. The oxygen consumption in the testing bottle was counted via a flow cell unit that opened if negative pressure is induced by CO_2 absorption. The biodegradability was estimated using the following equation:

$$\text{Biodegradability (\%)}, D_t = \frac{BOD_{sample} - BOD_{blank}}{ThOD} * 100$$

Where BOD_{sample} (mg kg^{-1}) represents the amount of oxygen consumption from a testing bottle at the time t, BOD_{blank} (mg kg^{-1}) is the amount of oxygen consumption from the blank at time t, and ThOD (mg kg^{-1}) is the maximum amount of oxygen consumption that could be theoretically calculated based on the chemical structure of the test materials.

Monomer synthesis

Synthesis of methyl vanillate and methyl 4-hydroxybenzoate

Synthesis of methyl vanillate was performed using a modified procedure found in the literature.[43] To a 500 mL round bottom flask, vanillic acid (25.0 g, 149 mmol), methanol (300 mL), and concentrated sulfuric acid (1.5 mL) were added at room temperature. The reaction mixture was refluxed overnight. Afterward, the reaction mixture was cooled to room temperature and concentrated *in vacuo*. The residue was dissolved in 100 mL of EtOAc. The organic phase was washed with saturated $NaHCO_3$ solution (100 mL) and brine (3 × 100 mL), dried over sodium sulfate, suction filtered, and concentrated *in vacuo*, yielding a white solid as methyl vanillate (25.2 g, 93%).

Synthesis of methyl 4-(2-hydroxyethoxy) benzoate [MEB] monomer

Methyl 4-hydroxybenzoate (20.7 g, 136 mmol, 1.00 eq.), ethylene carbonate (12.6 g, 143 mmol, 1.05 eq.), and potassium carbonate (1.87 g, 13.6 mmol, 0.10 eq.) was dissolved in DMSO (80 mL). The reaction mixture was stirred for 10 min and then heated to 100 °C under nitrogen for 3 h. After cooling to room temperature, DCM (400 mL) was added. The organic phase was washed with sodium hydroxide solution (0.50 M, 3 × 200 mL) and brine (3 × 200 mL), dried over sodium sulfate, suction filtered, and concentrated *in vacuo*, yielding a white solid as MEB (22.5 g, 84%). ^{1}H NMR (400.13 MHz, $CDCl_3$, δ, ppm): 3.87 (s, 3H, CO-OCH_3), 3.98 (t, 2H, J = 4.6 Hz, -CH_2-OH), 4.12 (t, 2H, J = 4.6 Hz, -O-CH_2-), 6.92 (d, 2H, J = 9.0 Hz, O-Ar-*H*), 7.98 (d, 2H, J = 9.0 Hz, MeOOC-Ar-*H*). ^{13}C NMR (100.61 MHz, $CDCl_3$, δ, ppm): 55.0, 61.4, 69.5, 114.3, 123.1, 131.7, 162.5, 166.9. T_m =66 °C (DSC). $T_{d,5\%}$= 147 °C (TGA). HRMS (ESI$^+$, m/z): [M+H$^+$] calculated for $C_{10}H_{13}O_4^+$: 197.0814, found 197.0811.

Synthesis of the methyl 4-(2-hydroxyethoxy) vanillate [MEV] monomer

Methyl vanillate (20.1 g, 110 mmol, 1.00 eq.), ethylene carbonate (9.70 g, 110 mmol, 1.00 eq.), and potassium carbonate (1.50 g, 11 mmol, 0.10 eq.) was dissolved in DMSO (80 mL). The reaction mixture was stirred for 10 min and then heated to 100 °C under nitrogen for 5 h. After cooling to room temperature, DCM (400 mL) was added. The organic phase was washed with sodium hydroxide solution (0.50 M, 3 × 200 mL) and brine (3 × 200 mL), dried over sodium sulfate, suction filtered, and concentrated *in vacuo*, yielding a white solid as MEV (19.4 g, 77 %).^{1}H NMR (400.13 MHz, $CDCl_3$, δ, ppm): 3.88 (s, 3H, CO-OCH_3), 3.89 (s, 3H, -O-CH_3), 3.99 (t, 2H, J = 4.6 Hz, -CH_2-OH), 4.16 (t, 2H, J = 4.6 Hz, O-CH_2-), 6.89 (d, 1H, J = 8.4 Hz, Ar-*H*), 7.54 (d, 1H, J = 2.0 Hz, Ar-H), 7.64 (d,d 1H, J = 8.4, 2.0 Hz, Ar-H). ^{13}C NMR (100.61 MHz, $CDCl_3$, δ, ppm): 52.1, 56.1, 61.2, 70.8, 76.8, 77.2, 77.5, 112.5, 123.4, 123.62, 149.1, 152.2, 166.9. HRMS (ESI$^+$, m/z): [M+H$^+$] calculated for $C_{11}H_{15}O_5^+$: 227.0919, found 227.0916.

Polymer synthesis

Poly(1,4-butylene adipate-co-4-(2-hydroxyethoxy) vanillate) [PBA(EV)] and Poly(1,4-butylene adipate-co-4-(2-hydroxyethoxy) benzoate) [PBA(EB)]

The synthesis of $PBA(EV)_{50}$ is described as a typical example. The polymerization to produce $PBA(EV)_{50}$ was performed in a 100 mL three-necked round-bottom flask equipped with a mechanical stirrer, a nitrogen inlet, and a 100 mL collection flask, which was connected to the vacuum outlet. To the flask was added MEV (5.65 g, 25.0 mmol, 1.00 eq.), BDO (2.53 g, 28.0 mmol, 1.13 eq.), DA (4.35 g, 25.0 mmol, 1.00 eq.) and DBTO (62.3 mg, 0.250 mmol). The reaction mixture was degassed three times by being subjected to a high vacuum for 20 min, followed by being backfilled with nitrogen. To start the polymerization, the reaction mixture was heated to 130 °C with mechanical stirring under N_2 for 2 h. Then, the temperature was increased to 150 °C with a steady stream of N_2 for another 2 h. Over the course of the reaction, methanol was distilled as by-product and collected in the collection flask. The conversion was monitored by ^{1}H NMR analysis, and the complete transesterification was observed after 4 h. Afterwards, the second step (polycondensation) was initiated at 150 °C over the period of 30 min with vacuum (~ 0.5 mbar). Afterwards, the reaction mixture was gradually heated to 200 °C for 6 h. For $PBA(EV)_{75}$, the final temperature was driven to 230 °C due to the high melting temperature. Afterward, the reaction mixture was cooled to room temperature and dissolved in a minimum volume of $CHCl_3$ (~50 mL). For $PBA(EV)_{75}$, HFIP (50 mL) was used instead of $CHCl_3$. The resulting solution was added dropwise to a vigorously stirred cold methanol solution (500 mL). The polymer was collected by filtration, washed three times with cold methanol, and dried under vacuum at 50 °C overnight to give a beige powder of $PBA(EV)_{50}$ (6.86 g, 68 %). ^{1}H NMR (400.13 MHz, $CDCl_3$, δ, ppm): 1.61 – 1.95 (m, 8H, –$CH_2C\mathit{H}_2CH_2$–), 2.34 (d, J = 19.1 Hz, 4H, –$C\mathit{H}_2COO$–), 3.85 – 3.89 (m, 3H, –OCH_3), 4.12 (m, 4H, –$C\mathit{H}_2O$–, A–*B*), 4.27 (br, 2H, –

OCH_2CH_2O–, A–*EV*), 4.29 –4.40 (m, 4H, –CH_2O–, EV-*B*), 4.42 (t, *J* = 4.9 Hz, 2H, –OCH_2CH_2O–, EV–*EV*), 4.46 (br, 2H, –OCH_2CH_2O–, A–*EV*), 4.69 (t, *J* = 5.0 Hz, 2H, –OCH_2CH_2O–, EV–*EV*), 6.85 – 6.99 (m, 1H, ArH), 7.55 (s, 1H, ArH), 7.61 –7.69 (m, 1H, ArH). ^{13}C NMR (100.61 MHz, $CDCl_3$, δ, ppm): 24.4, 25.3, 33.8, 56.0, 62.4, 62.9, 63.9, 64.3, 66.9, 112.2, 112.7, 123.3, 123.7, 149.1, 151.9, 166.2, 173.2. SEC ($CHCl_3$, 1 mL min^{-1}), M_n= 18.4 kg mol^{-1}, *Đ* =2.39.

The other PBA(EV) and PBA(EB) copolyesters were synthesized using a similar protocol, and the conditions are summarized in Table 1.

PBA(EV)$_{25}$. ^{1}H NMR (400.13 MHz, $CDCl_3$, δ, ppm): 1.56 – 1.95 (m, 8H, –$CH_2CH_2CH_2$–), 2.24 – 2.39 (d, *J* = 19.1 Hz, 4H, –CH_2COO–), 3.84 – 3.91 (m, 3H, –OCH_3), 4.03 – 4.15 (m, 4H, –CH_2O–, A–*B*), 4.28 (dt, *J* = 21.1, *J* = 5.5 Hz, 2H, –OCH_2CH_2O–, A–*EV*), 4.34 – 4.43 (m, 4H, –CH_2O–, EV–*B*), 4.45 (t, *J* = 4.8 Hz, 2H, –OCH_2CH_2O–, EV–*EV*), 4.46 (br, 2H, –OCH_2CH_2O–, A–*EV*), 4.67 (t, *J* = 5.0 Hz, 2H, –OCH_2CH_2O–, EV–*EV*), 6.85 – 6.97 (m, 1H, ArH), 7.53 (s, 1H, ArH), 7.59 – 7.65 (m, 1H, ArH). ^{13}C NMR (100.61 MHz, $CDCl_3$, δ, ppm): 24.8, 25.2, 33.8, 56.0, 62.3, 63.7, 66.8, 112.2, 112.6, 123.2, 123.4, 149.0, 151.9, 166.1, 173.2. SEC ($CHCl_3$, 1 mL min^{-1}), M_n= 11.5 kg mol^{-1}, *Đ* = 2.44.

PBA(EV)$_{60}$. ^{1}H NMR (400.13 MHz, $CDCl_3$, δ, ppm): 1.57 – 1.97 (m, 8H, –$CH_2CH_2CH_2$–), 2.23 – 2.41 (m, 4H, –CH_2COO–), 3.84 – 3.91 (m, 3H, –OCH_3), 4.03 – 4.17 (m, 4H, –CH_2O–, A–*B*), 4.26 (br, 2H, –OCH_2CH_2O–, A–*EV*), 4.28 – 4.39 (m, 4H, –CH_2O–, EV–*B*), 4.41 (t, *J* = 5.1 Hz, 2H, –OCH_2CH_2O–, EV–*EV*), 4.46 (br, 2H, –OCH_2CH_2O–, A–*EV*), 4.67 (t, *J* = 5.0 Hz, 2H, –OCH_2CH_2O–, EV–*EV*), 6.82 – 6.98 (m, 1H, ArH), 7.53 (s, 1H, ArH), 7.60 – 7.67 (m, 1H, ArH). ^{13}C NMR (100.61 MHz, $CDCl_3$, δ, ppm): 24.3, 25.2, 33.6, 56.0, 62.3, 62.9, 63.7, 64.3, 66.8, 67.1, 112.13, 112.8, 123.2, 123.4, 149.0, 151.9, 166.0, 173.1. SEC ($CHCl_3$, 1 mL min^{-1}), M_n= 14.8 kg mol^{-1}, *Đ* = 2.43.

PBA(EV)$_{75}$. ^{1}H NMR (400.13 MHz, $CF_3COOD/CDCl_3$ *(8:2 v/v)*, δ, ppm): 1.59 – 2.02 (m, 8H, –$CH_2CH_2CH_2$–), 2.43 (t, *J* = 3.6 Hz, 4H, –C*H*$_2$COO–), 3.88 – 3.96 (m, 3H, –OCH_3), 4.12 – 4.25 (m, 4H, –C*H*$_2$CH$_2$O–, A–*B*), 4.31 (br, 2H, –OC*H*$_2$CH$_2$O–, A–EV), 4.36 – 4.49 (m, 4H, –C*H*$_2$CH$_2$O–, EV–*B*), 4.52 (br, 2H, –OCH$_2$C*H*$_2$O–, A–*EV*), 4.72 (br, 2H, –OCH$_2$C*H*$_2$O–, EV–*EV*), 6.87 – 7.02 (m, 1H, ArH), 7.58 (s, 1H, ArH), 7.67 – 7.75 (m, 1H, ArH). ^{13}C NMR (100.61 MHz, $CDCl_3$, δ, ppm): 23.8, 25.2, 33.6, 56.0, 63.2, 63.6, 65.3, 65.6, 66.6, 66.9, 112.1, 112.9, 122.5, 124.6, 148.4, 152.1, 167.9, 175.9.

PBA(EB)$_{25}$. ^{1}H NMR (400.13 MHz, $CDCl_3$, δ, ppm): 1.56 – 1.96 (m, 8H, –CH$_2$C*H*$_2$CH$_2$–), 2.26 – 2.44 (m, 4H, –C*H*$_2$COO–), 4.05 – 4.16 (m, 4H, –C*H*$_2$CH$_2$O–, A–*B*), 4.21 (t, *J* = 4.8 Hz, 2H, –OC*H*$_2$CH$_2$O–, A–*EB*), 4.28 – 4.38 (m, 4H, –C*H*$_2$CH$_2$O–, EB–*B*), 4.43 (t, *J* = 4.8 Hz, 2H, –OCH$_2$C*H*$_2$O–, A–*EB*), 4.64 (t, *J* = 4.7 Hz, 2H, –OCH$_2$C*H*$_2$O–, EB–*EB*), 6.89 – 6.98 (m, 2H, ArH), 8.0 (br, 2H, ArH). ^{13}C NMR (100.61 MHz, $CDCl_3$, δ, ppm): 24.3, 25.2, 33.7, 62.3, 62.7, 63.7, 64.1, 65.9, 66.1, 114.1, 123.1, 131.5, 162.1, 166.1, 173.2. SEC ($CHCl_3$, 1 mL min^{-1}), M_n= 15.7 kg mol^{-1}, *Đ* =2.22.

PBA(EB)$_{50}$. ^{1}H NMR (400.13 MHz, $CDCl_3$, δ, ppm): 1.57 – 1.98 (m, 8H, –CH$_2$C*H*$_2$CH$_2$–), 2.26 – 2.43 (m, 4H, –C*H*$_2$COO–), 4.03 – 4.18 (m, 4H, –C*H*$_2$CH$_2$O–, A–*B*), 4.22 (br, 2H, –OC*H*$_2$CH$_2$O–, A–*EB*), 4.29 – 4.40 (m, 4H, –C*H*$_2$CH$_2$O–, EB–*B*), 4.44 (br, 2H, –OCH$_2$C*H*$_2$O–, A–*EB*), 4.66 (t, *J* = 4.9 Hz, 2H, –OCH$_2$C*H*$_2$O–, EB–*EB*), 6.94 (dd, 2H, *J* = 16.7, *J* = 8.3, Hz ArH), 8.0 (br, 2H, ArH). ^{13}C NMR (100.61 MHz, $CDCl_3$, δ, ppm): 24.3, 25.2, 33.7, 62.3, 62.7, 63.7, 64.0, 65.9, 66.1, 114.1, 123.1, 131.5, 162.1, 166.1, 173.2. SEC ($CHCl_3$, 1 mL min^{-1}), M_n= 12.9 kg mol^{-1}, *Đ* =2.57.

PBA(EB)$_{60}$. ^{1}H NMR (400.13 MHz, $CDCl_3$, δ, ppm): 1.61 – 1.96 (m, 8H, –CH$_2$C*H*$_2$CH$_2$–), 2.28 – 2.43 (m, 4H, –C*H*$_2$COO–), 4.07 – 4.17 (m, 4H, –C*H*$_2$CH$_2$O–, A–*B*), 4.21 (br, 2H, –OC*H*$_2$CH$_2$O–, A–*EB*), 4.29 – 4.40 (m, 4H, –C*H*$_2$CH$_2$O–, EB–*B*), 4.44 (br, 2H, –OCH$_2$C*H*$_2$O–,

A–*EB*), 4.65 (t, J = 4.9 Hz, 2H, –OCH_2CH_2O–, EB–*EB*), 6.94 (dd, 2H, J = 17.3, J = 8.3 Hz, ArH), 8.0 (br, 2H, ArH). ^{13}C NMR (100.61 MHz, $CDCl_3$, δ, ppm): 24.1, 25.5, 33.6, 62.3, 62.7, 63.7, 64.2, 65.9, 66.1, 114.1, 123.1, 131.5, 162.1, 165.9, 173.0. SEC ($CHCl_3$, 1 mL min^{-1}), M_n= 17.1 kg mol^{-1}, $Đ$ =2.8.

PBA(EB)$_{75}$. ^1H NMR (400.13 MHz, $CDCl_3$, δ, ppm): 1.59 – 1.96 (m, 8H, –$CH_2CH_2CH_2$–), 2.30 – 2.41 (m, 4H, –CH_2COO–), 4.07 – 4.18 (m, 4H, –CH_2CH_2O–, A–*B*), 4.22 (br, 2H, –OCH_2CH_2O–, A–*EB*), 4.29 – 4.40 (m, 4H, –CH_2CH_2O–, EB–*B*), 4.44 (br, 2H, –OCH_2CH_2O–, A–*EB*), 4.66 (t, J = 4.9 Hz, 2H, –OCH_2CH_2O–, EB–*EB*), 6.94 (dd, 2H, J = 19.0, J = 8.3 Hz, ArH), 8.0 (br, 2H, ArH). ^{13}C NMR (100.61 MHz, $CDCl_3$, δ, ppm): 24.1, 25.5, 33.6, 62.3, 62.7, 63.8, 64.2, 65.9, 66.1, 114.1, 122.5, 131.7, 162.4, 166.1, 173.0. SEC ($CHCl_3$, 1 mL min^{-1}), M_n= 19.0 kg mol^{-1}, $Đ$ =2.58.

Characterization of the monomers, MEV and MEB

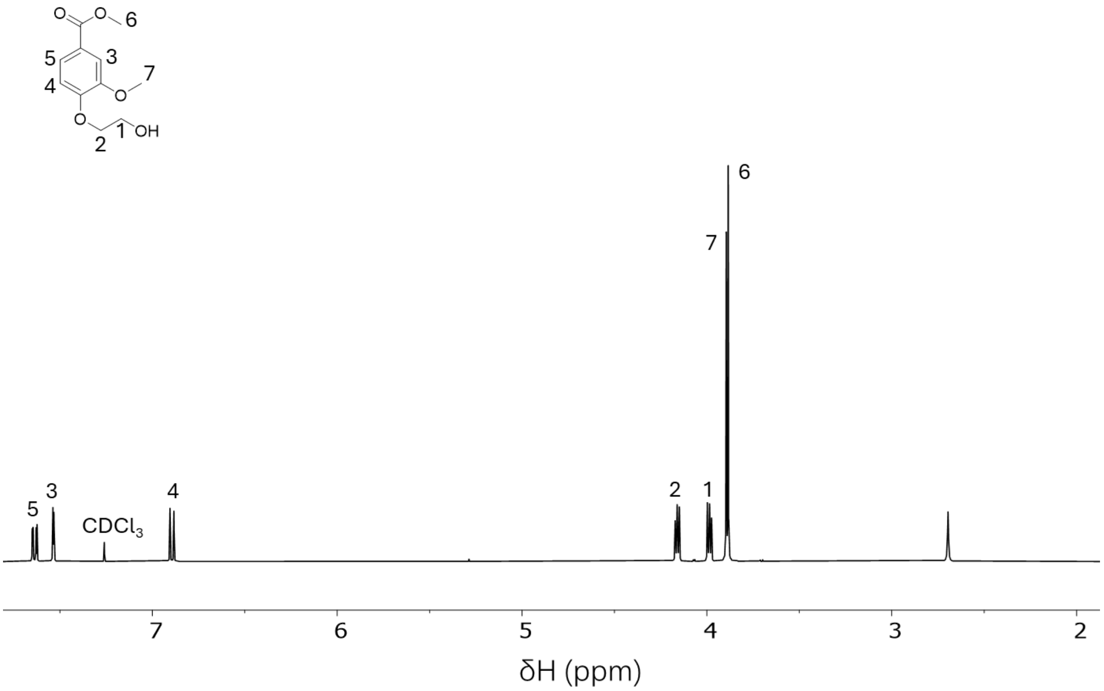

Figure S1. ^{1}H NMR (400.13 MHz, $CDCl_3$) spectrum of monomer MEV.

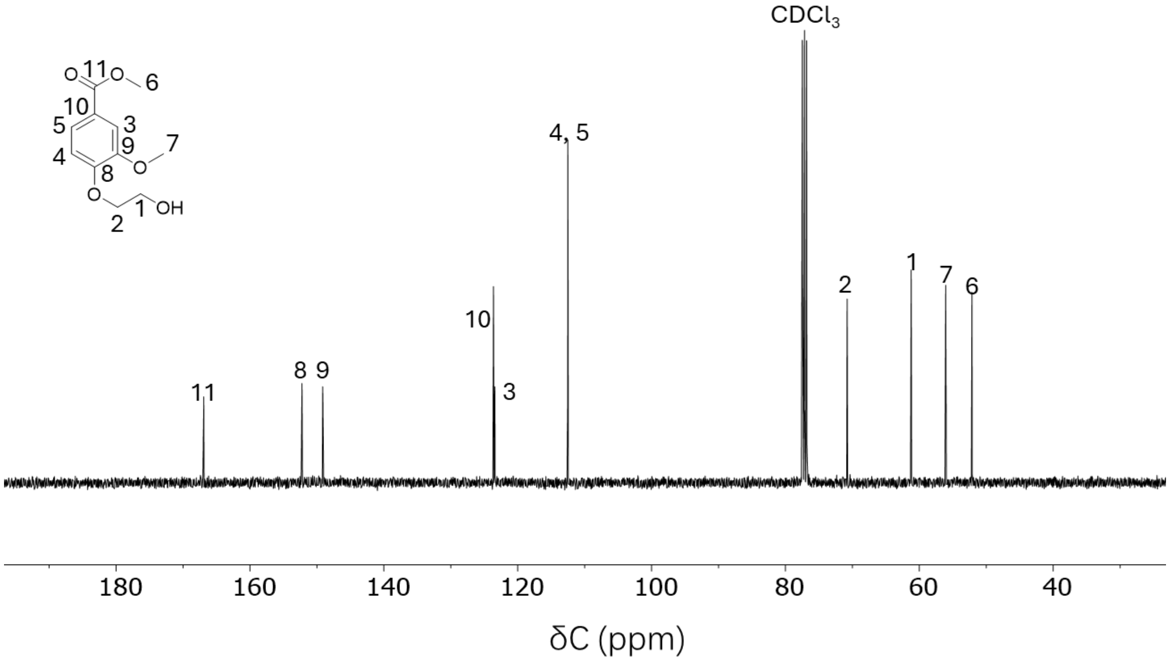

Figure S2. ^{13}C NMR (100.61 MHz, $CDCl_3$) spectrum of monomer MEV.

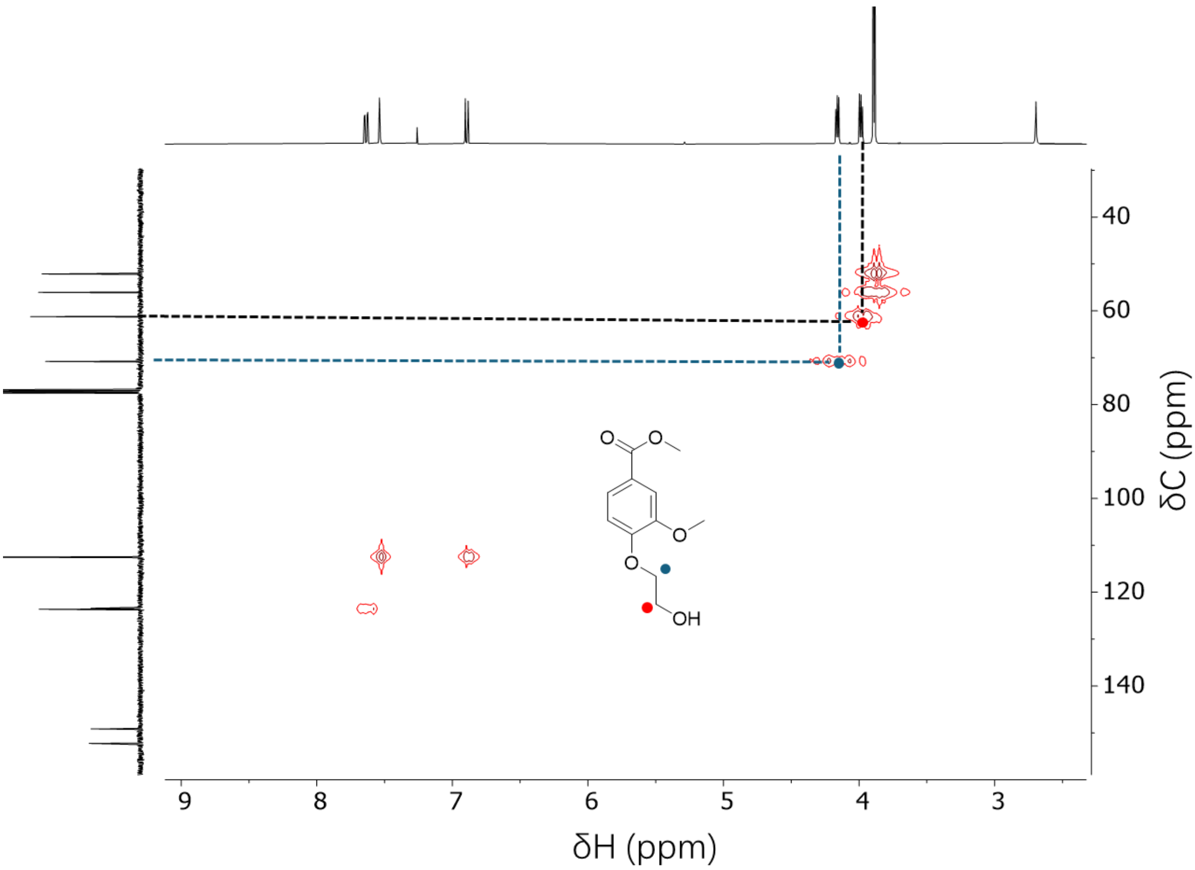

Figure S3. HSQC NMR spectrum of monomer MEV in $CDCl_3$.

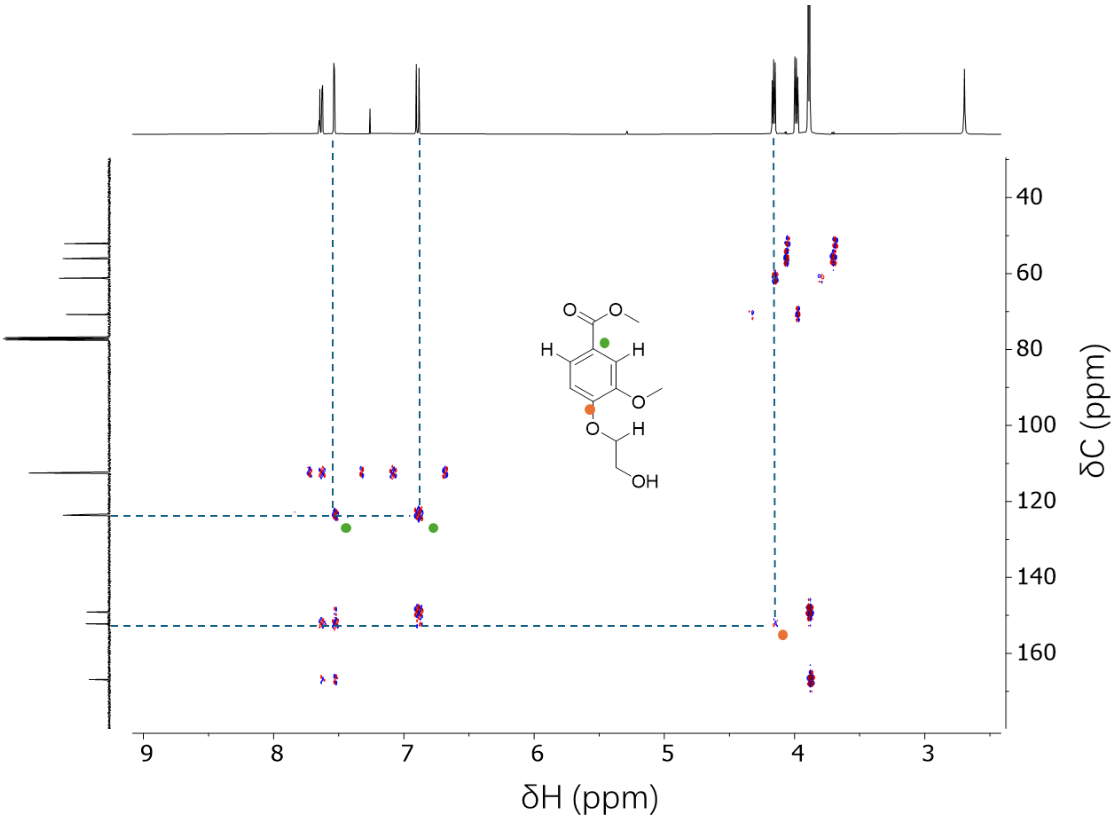

Figure S4. HMBC NMR spectrum of monomer MEV in $CDCl_3$.

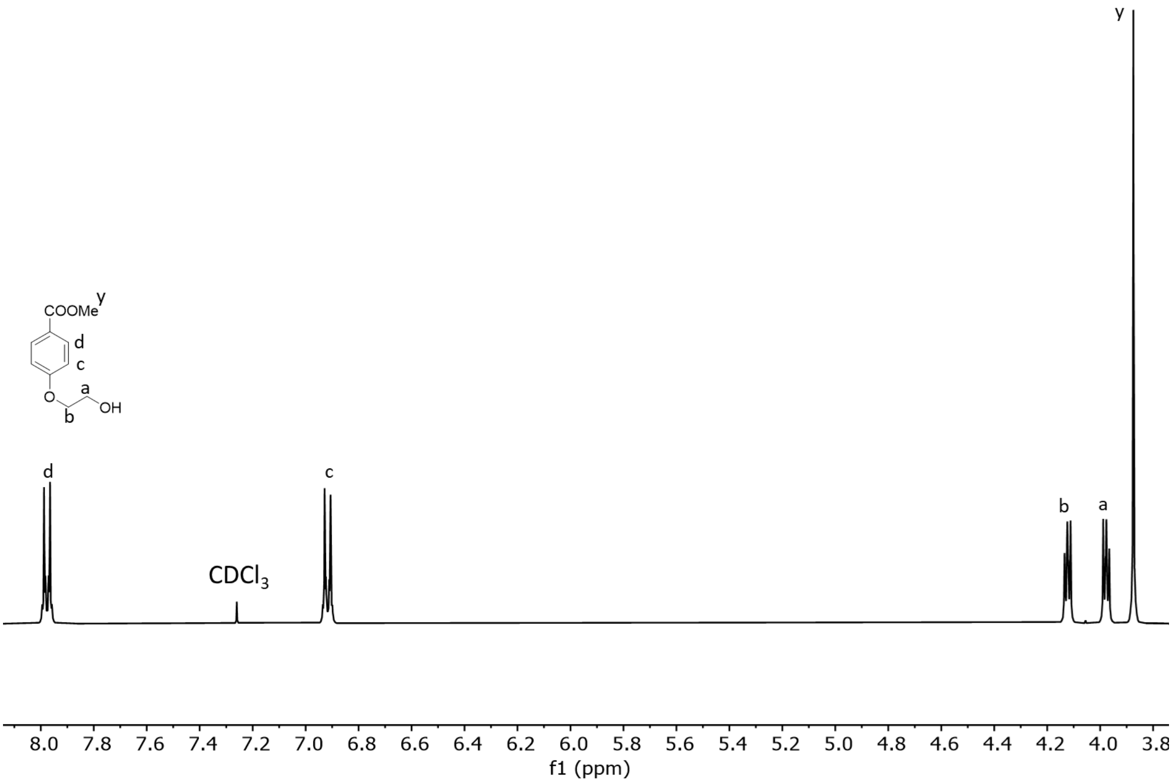

Figure S5. ^{1}H NMR (400.13 MHz, $CDCl_3$) spectrum of monomer MEB.

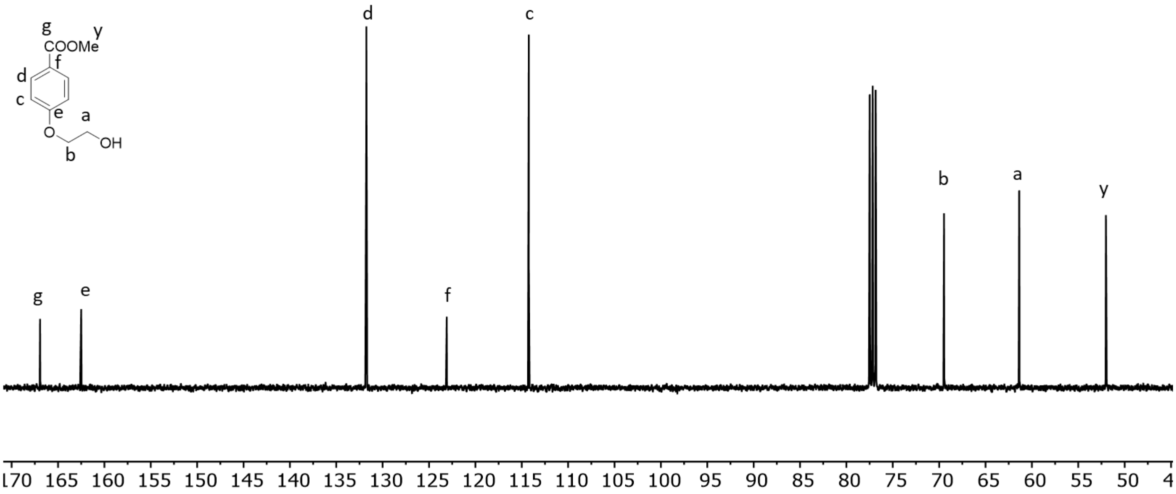

Figure S6. ^{13}C NMR (100.61 MHz, $CDCl_3$) spectrum of monomer MEB.

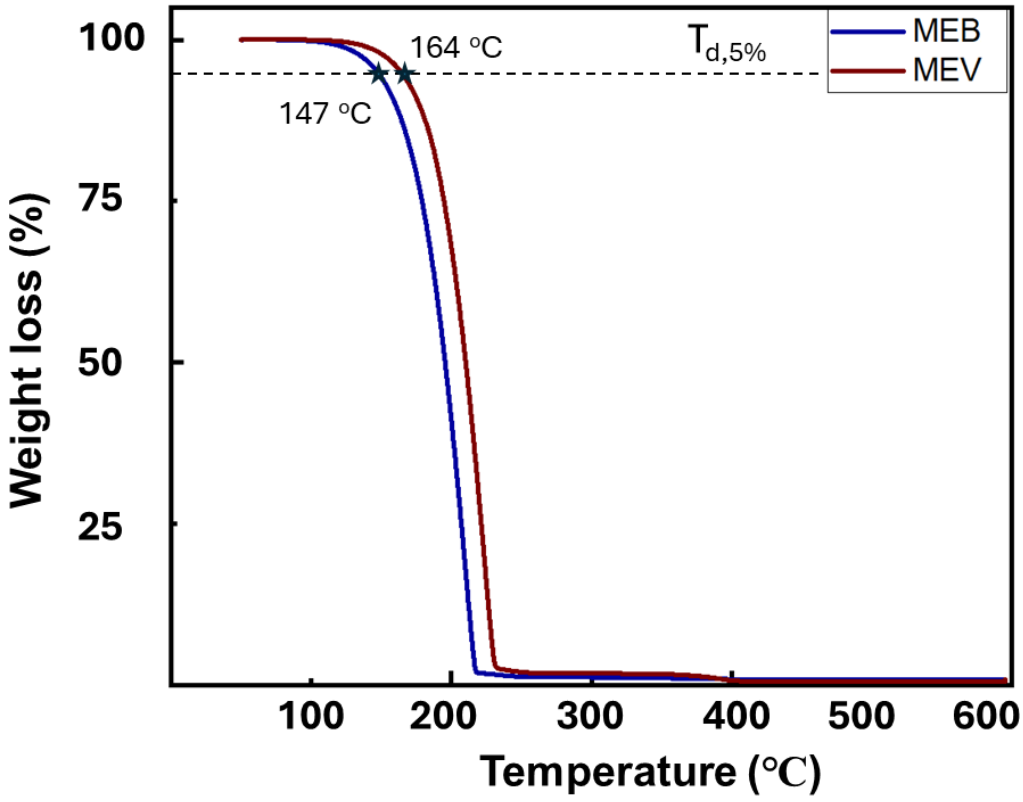

Figure S7. TGA weight loss curves of MEV and MEB monomers.

Characterization of the copolyesters, PBA(EV)s and PBA(EB)s

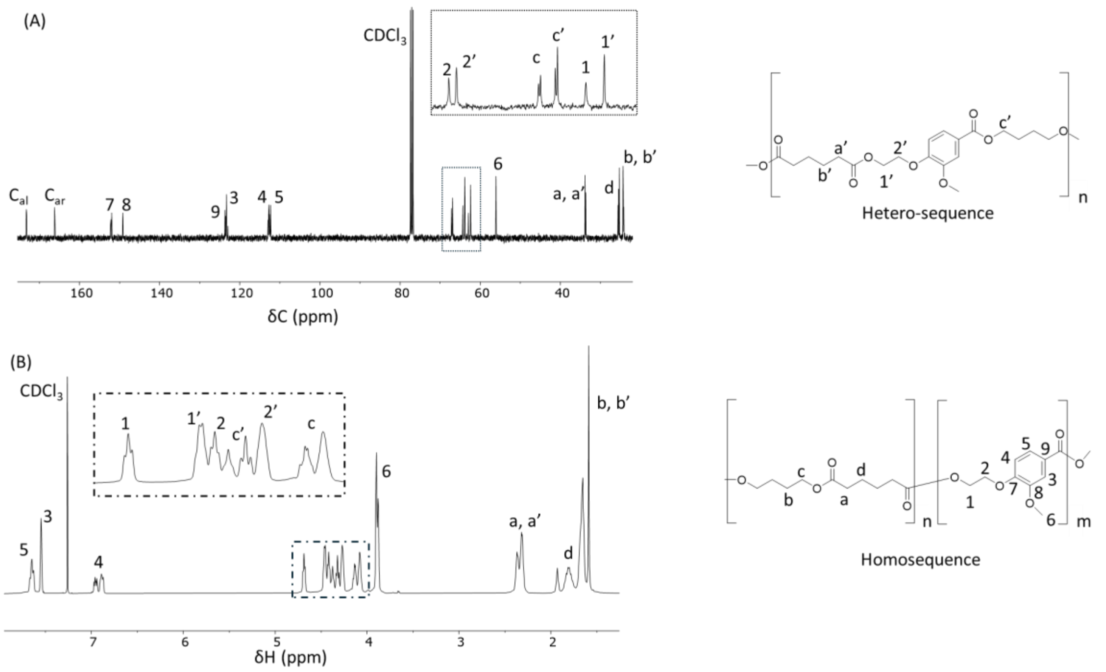

Figure S8. 1H NMR and 13C NMR of PBA(EV)$_{50}$ with complete peak assignment record in CDCl$_3$.

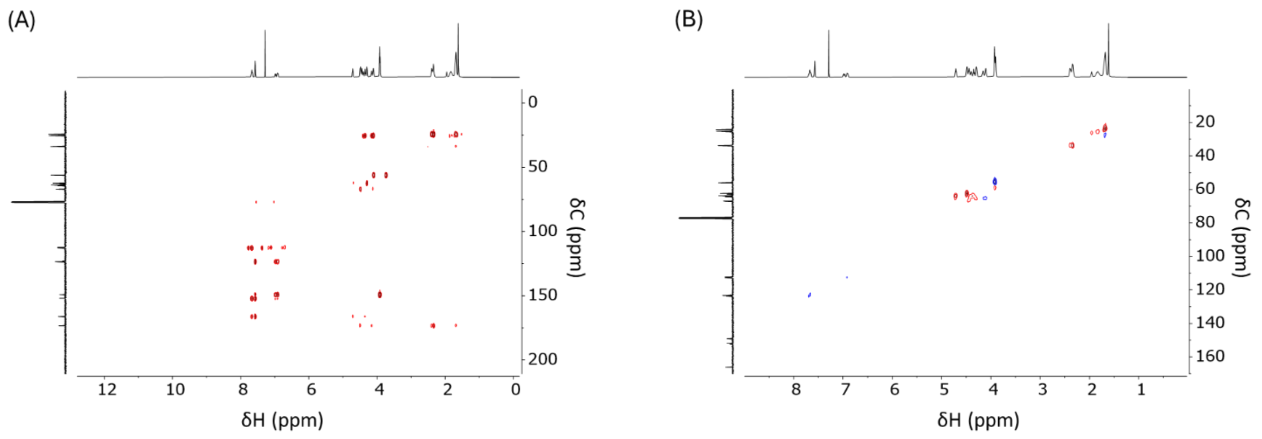

Figure S9. 2D NMR (A) HMBC and (B) HSQC spectra of PBA(EV)$_{50}$.

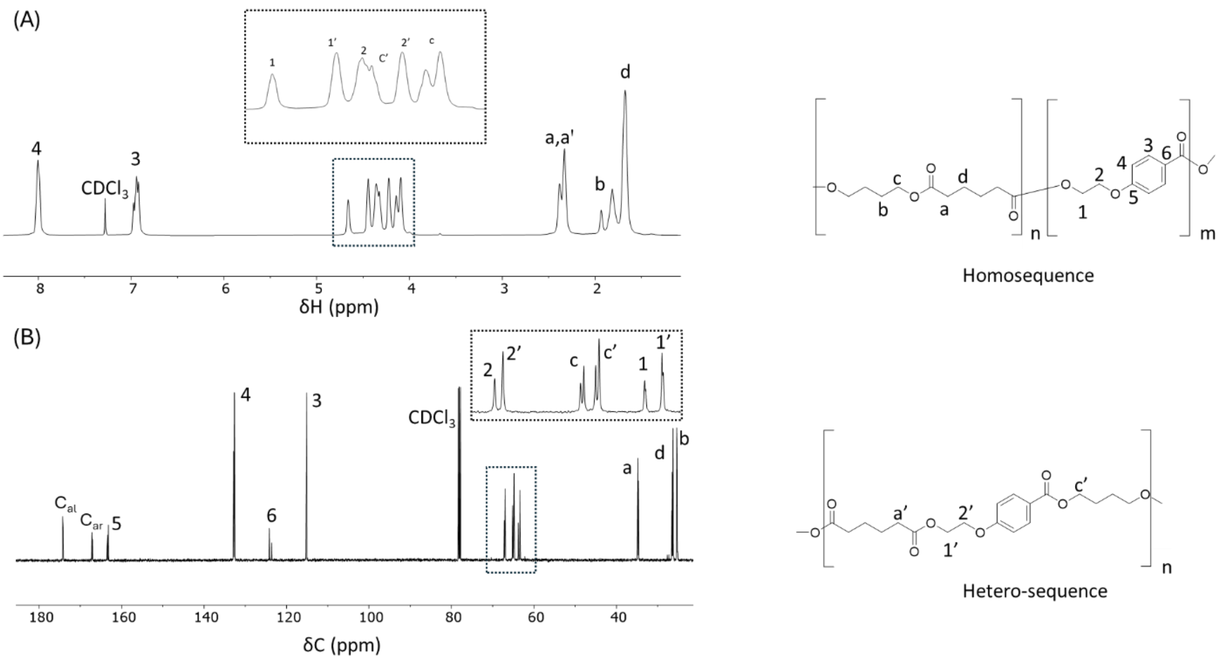

Figure S10. ^{1}H NMR (A) and ^{13}C NMR (B) spectra with complete peak assignment of PBA(EB)$_{50}$ recorded in $CDCl_3$.

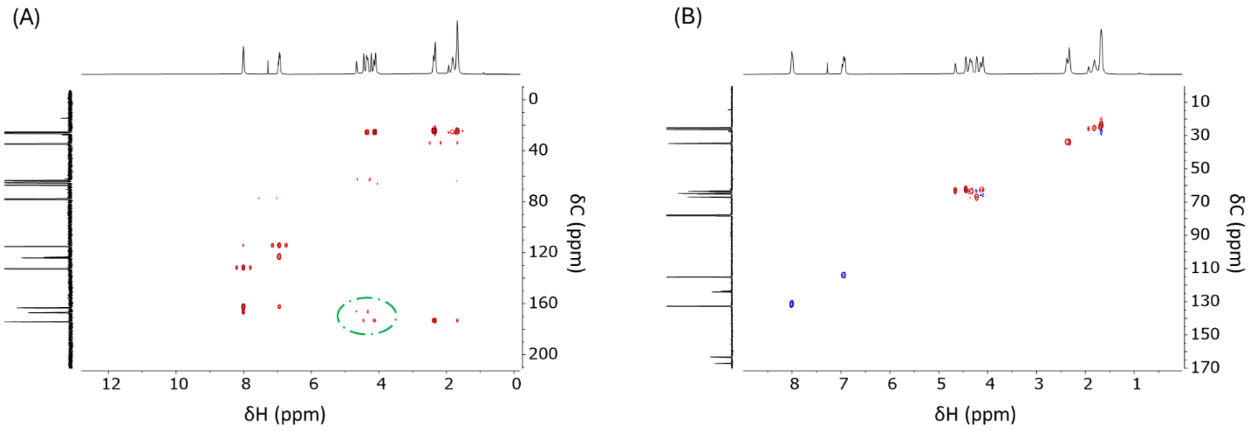

Figure S11. 2D NMR (A) HMBC and (B) HSQC spectra of PBA(EB)$_{50}$.

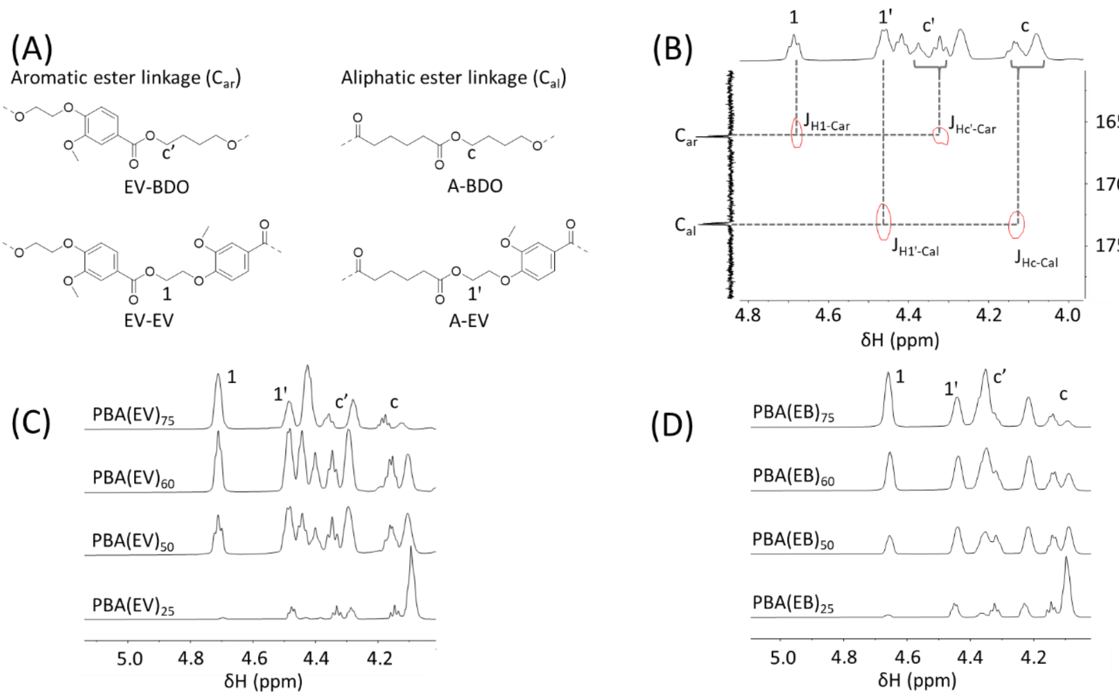

Figure S12. (A) Four possible dyads in PBA(EV) copolyesters. (B) Selected region of the 2D NMR HMBC spectrum of PBA(EV)$_{50}$. Enlarged region (4.0-5.0 ppm) of the ^{1}H NMR spectra of (C) PBA(EV) and (D) PBA(EB) copolyesters with different compositions. All NMR spectra were acquired in $CDCl_3$ except PBA(EV)$_{75}$, recorded in $CDCl_3$ and TFA-d (8:2 v/v).

Solution-casting film

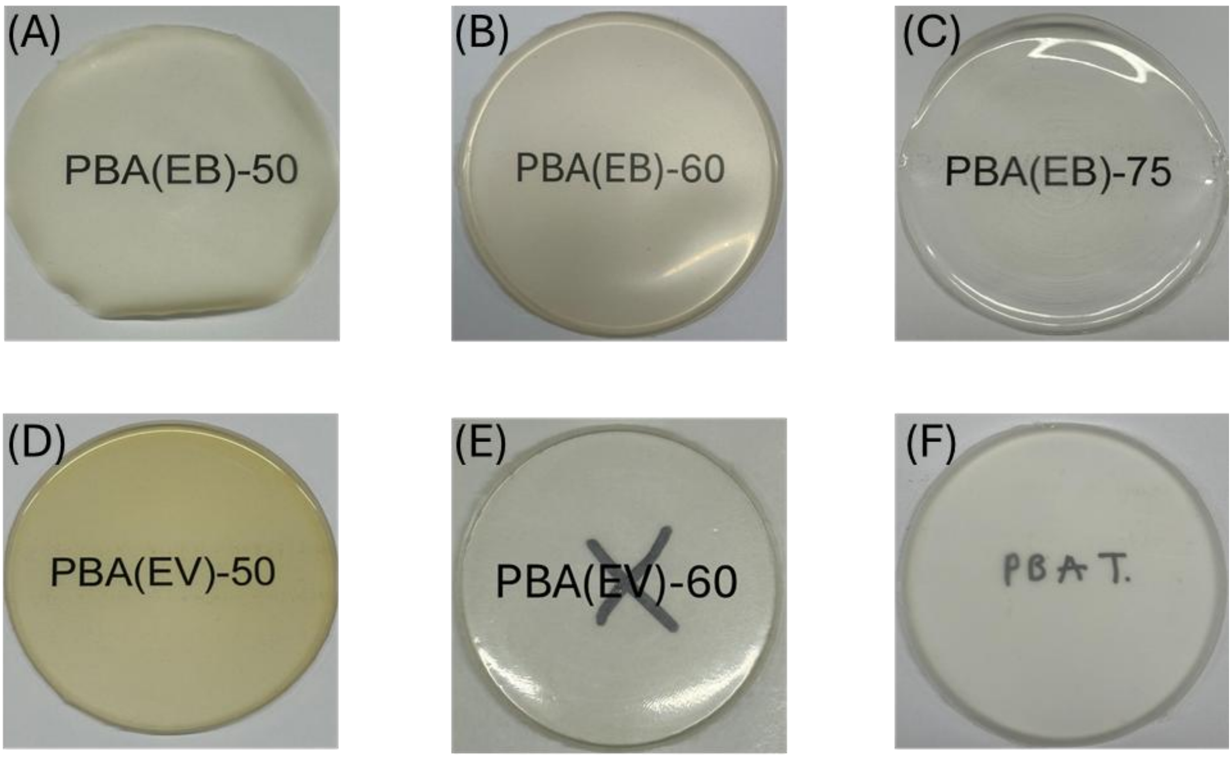

Figure S13. Solvent-cast films of (A) PBA(EB)$_{50}$, (B) PBA(EB)$_{60}$, (C) PBA(EB)$_{75}$, (D) PBA(EV)$_{50}$ (E) PBA(EV)$_{60}$ (F) PBAT.

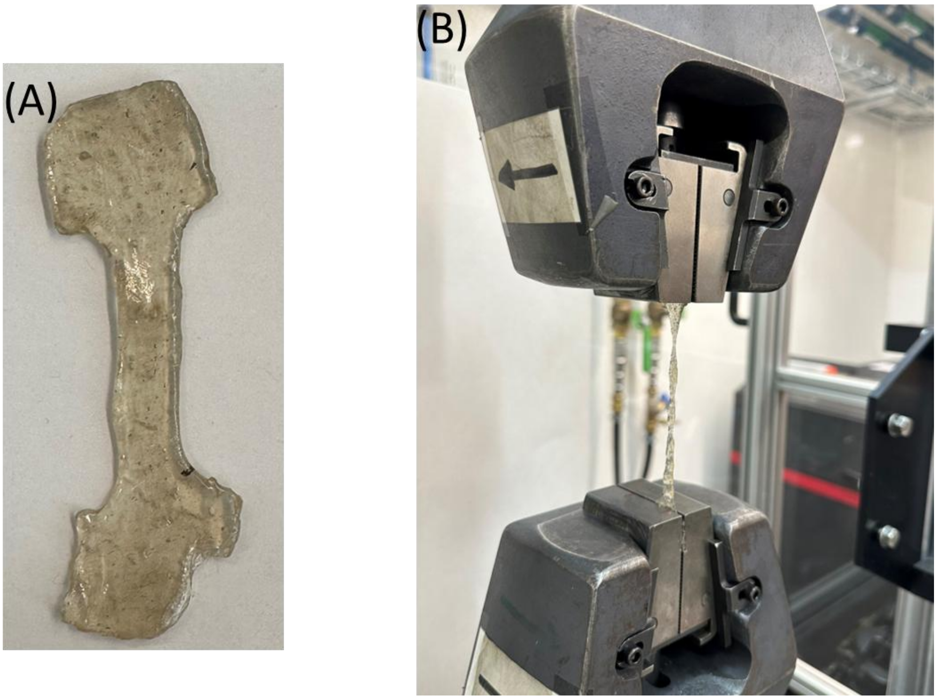

Figure S14. (A) Deformed PBA(EB)$_{50}$ specimen after hot-pressed. (B) Unsuccessful tensile testing of PBA(EB)$_{50}$ specimen due to lack of mechanical rigid.

of (C) PBA(EV) and (D) PBA(EB) copolyesters with different compositions. All NMR spectra were acquired in $CDCl_3$ except PBA(EV)$_{75}$, recorded in $CDCl_3$ and TFA-d (8:2 v/v).

Table S1. Solvent screening for the synthesis of monomer MEV[a]

	Solvent	MEV (%)	Self-condensation (PEV) (%)	Side reactions (%)
Recommended[b]	Solvent-free	14	5	63
	i-BuOH	5	-	79
	Ethylene glycol	-	-	66
	Anisole	6	-	-
	MIBK	10	-	-
	MIBK (+18-Crown-6)*	8	-	-
	MIBK (DBU)**	-	-	-
	Cyclohexanone	-	-	-
Non-recommended[b]	DMF	50	-	-
	DMSO	81	-	-
	DMSO (2mL)	84	-	-
	DMSO (1mL)	81	> 10[d]	-

[a] *Reaction condition: 1 g of methyl vanillate in a solvent (6 mL) with 10 mol% K_2CO_3 as the catalyst at 100 ℃ for 5 hours. The conversion was estimated by ^1H NMR spectroscopy analysis of the crude reaction mixture after 5h.*
[b]*According to the recommendation of CHEM21 solvent guidelines.*[40]
[c]*Side reactions include transesterification and ring-opening polymerization of cyclic carbonate ring.*
[d] *The oligomerization was underestimated due to the precipitation of the oligomer in the crude reaction mixture.*
* *18-crown-6 was added to increase the solubility of K_2CO_3 in the organic solvent*
** *10 mol% DBU was used as the catalyst instead of K_2CO_3*

Green metrics: E factor and atom economy

The greenness assessment of the synthetic procedures was carried out based on two widely accepted green metrics: environmental factor (E factor) and atom economy (AE). Simple E factor (sEF), which excludes solvent usage in the reaction, and complete E factor (cEF) that include solvents without recycling were also calculated. The solvents/chemicals used in the work-up were ruled out of the calculation to allow meaningful conclusions. Nonetheless, the work-up step must be revisited at the manufacturing stage. The equations for all green metrics were listed:[49]

$$sEF = \frac{\sum m\ (raw\ materials) + \sum m\ (catalysts) - m(desired\ product)}{m(desired\ product)}$$

$$cEF = \frac{\sum m\ (raw\ materials) + \sum m\ (catalysts) + \sum m\ (solvents) - m(desired\ product)}{m(desired\ product)}$$

$$AE = \frac{M\ (\ product)}{\sum M(reactants)} \times 100\%$$

Table S2. Green metric calculations of monomer syntheses

	Input	Eq	Molar mass	Weight	Output weight	Yield	AE	sEF	E	Solvent contribution
		Mol	g mol^{-1}	g	g	%	%			%
**MEV**										
**Step1**	Vanillic acid	**0.15**	**168**	**25**	**25.18**	**93**	**91**	**0.29**	**9.54**	**97**
	Methanol	**7.42**	**32**	**237.6**						
	Sulfuric acid	**0.03**	**98**	**2.85**						
	Methyl vanillate	**0.14**	**182**							
**Step2**	Methyl vanillate	**0.11**	**182**	**20.1**	**19.4**	**77**	**81**	**0.58**	**7.95**	**93**
	Ethylene carbonate	**0.11**	**88**	**9.1**						
	DMSO (solvent)	**1.83**	**78**	**143**						
	K_2CO_3	**0.01**	**138**	**1.5**						
	MEV	**0.09**	**226**							
**MEB**										
**Step1**	4-hydroxyl benzoic	**0.18**	**138**	**25**	**25.6**	**92**	**89**	**0.27**	**9.37**	**97**
	Methanol	**7.4**	**32**	**238**						
	Sulfuric acid	**0.03**	**98**	**2.85**						
	Methyl paraben	**0.17**	**152**							
**Step2**	Methyl paraben	**0.14**	**152**	**20.7**	**22.5**	**84**	**76**	**0.56**	**6.92**	**92**
	Ethylene carbonate	**0.14**	**88**	**12.6**						
	DMSO (solvent)	**1.83**	**78**	**143**						
	K_2CO_3	**0.01**	**138**	**1.87**						
	MEB	**0.12**	**196**							

Table S3. Effect of initial excess of diol on the polymerization of PBA(EV)$_{50}$.

Excess BDO (mol%)	Feed ratio MEV	DA	BDO	Mn kg mol^{-1}	Mw kg mol^{-1}	PDI
0	1.0	1.0	1.0	8.7	19.1	2.2
5	1.0	1.0	1.05	18.4	44.2	2.4
12.5	1.0	1.0	1.125	12.5	37.5	3.0

Table S4. Summary of tensile properties for the obtained copolyesters compared to that of PBAT.

	PBAT	PBA(EV)$_{50}$	PBA(EB)$_{50}$
E (MPa)	64 ± 4.2	56 ± 1.7	-
σ_s (Mpa)	9.6 ±0.6	10 ±0.2	-
ε_b (%)	89 ± 45	97 ± 29	-
